# Molybdenum-Catalyzed Enantioselective Sulfoxidation Controlled by a Nonclassical Hydrogen Bond between Coordinated Chiral Imidazolium-Based Dicarboxylate and Peroxido Ligands

**DOI:** 10.3390/molecules23071595

**Published:** 2018-06-30

**Authors:** Carlos J. Carrasco, Francisco Montilla, Agustín Galindo

**Affiliations:** Departamento de Química Inorgánica, Universidad de Sevilla, Aptdo. 1203, 41071 Sevilla, Spain; ccarrasco1@us.es

**Keywords:** sulfoxidation, asymmetric catalysis, molybdenum, hydrogen peroxide, Density Functional Theory

## Abstract

Chiral alkyl aryl sulfoxides were obtained by molybdenum-catalyzed oxidation of alkyl aryl sulfides with hydrogen peroxide as oxidant in mild conditions with high yields and moderate enantioselectivities. The asymmetry is generated by the use of imidazolium-based dicarboxylic compounds, HL^R^. The in-situ-generated catalyst, a mixture of aqueous [Mo(O)(O_2_)_2_(H_2_O)_n_] with HL^R^ as chirality inductors, in the presence of [PPh_4_]Br, was identified as the anionic binuclear complex [PPh_4_]{[Mo(O)(O_2_)_2_(H_2_O)]_2_(μ-L^R^)}, according to spectroscopic data and Density Functional Theory (DFT) calculations. A nonclassical hydrogen bond between one C–H bond of the alkyl R group of coordinated (L^R^)^−^ and one oxygen atom of the peroxido ligand was identified as the interaction responsible for the asymmetry in the process. Additionally, the step that governs the enantioselectivity was theoretically analyzed by locating the transition states of the oxido-transfer to PhMeS of model complexes [Mo(O)(O_2_)_2_(H_2_O)(κ^1^-*O*-L^R^)]^−^ (R = H, ^i^Pr). The ∆∆G^≠^ is ca. 0 kcal∙mol^−1^ for R = H, racemic sulfoxide, meanwhile for chiral species the ∆∆G^≠^ of ca. 2 kcal∙mol^−1^ favors the formation of (*R*)-sulfoxide.

## 1. Introduction

**Scheme 1:**
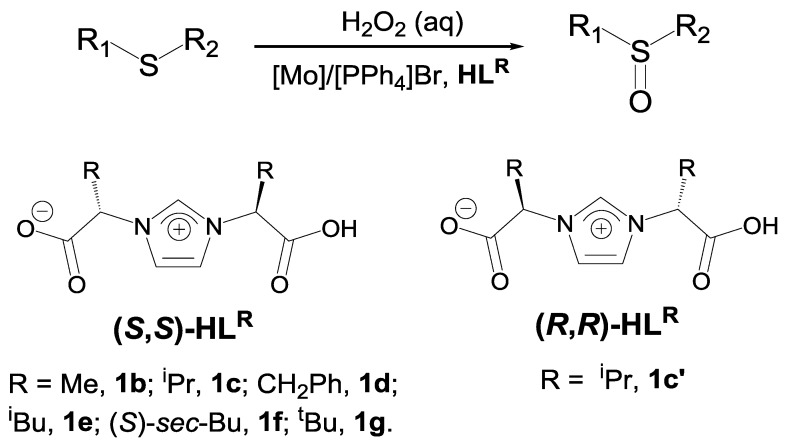
Enantioselective sulfoxidation with hydrogen peroxide in the presence of chiral inductors HL^R^.

The synthesis and use of enantiopure sulfoxides is a topic of extraordinary interest in asymmetric synthesis, asymmetric catalysis and in the pharmaceutical industry [1,2,3,4,5,6,7,8,9]. The enantioselective sulfoxidation of prochiral sulfides is one of the most challenging approaches to chiral sulfoxides, and catalyzed processes based on metal complexes [3,4,10,11,12,13,14,15,16] and metal-free systems [17] have been described in the literature. Molybdenum-catalyzed enantioselective sulfoxidations have been investigated [18,19,20,21,22,23,24,25] and, in general, the Mo catalysts provided results that are somewhat lower than those of other metals, as for example titanium [26,27,28,29,30,31,32] or vanadium [33,34,35,36,37,38,39,40,41] complexes. However, we have recently demonstrated that the use of the imidazolium-based dicarboxylic compound (*S*,*S*)-1-(1-carboxy-2-methylpropyl)-3-(1-carboxylate-2-methylpropyl)imidazolium (HL^iPr^ in Scheme 1), as inductor of chirality, in combination with oxidoperoxidomolybdenum complexes, afforded a system capable to achieve by kinetic resolution a value of 83% *ee* in the sulfoxidation of alkyl aryl sulfides [42]. This system is easily accessible, simple, environmentally friendly, and compatible with a green oxidant as aqueous hydrogen peroxide. In some cases, these advantages are not compatible with Ti or V catalysts. Following our recent research on Mo-catalyzed sulfoxidations [42,43,44], we report here the extension of our system [42] to other imidazolium-based dicarboxylic compounds, HL^R^, in order to improve its efficiency in the catalytic asymmetric oxidation of prochiral sulfides using aqueous hydrogen peroxide (Scheme 1). Moreover, spectroscopic data and Density Functional Theory (DFT) calculations have allowed identification of the nature of the molybdenum catalytic species, {[Mo(O)(O_2_)_2_(H_2_O)]_2_(μ-L^R^)}^−^, and the origin of the asymmetry in the sulfoxidation process.

## 2. Results and Discussion

### 2.1. Enantioselective Oxidation of Different Sulfides with Aqueous Hydrogen Peroxide Catalyzed by the System [Mo(O)(O_2_)_2_(H_2_O)_n_]/HL^R^/[PPh_4_]Br

The optimization of the reaction conditions were performed with the (*S*,*S*)-HL^iPr^ compound, **1c**, and methyl phenyl sulfide, and were previously communicated [42]. Chloroform was used as solvent (1 mL) with a 1:1:0.025:2 ratio of methyl phenyl sulfide:H_2_O_2_:Mo-complex:[PPh_4_]Br. Reactions were carried out in a micro-reactor, at 0 °C during 1 h, on 1 mmol scale. A solution of MoO_3_ (2.5% mmol) in aqueous hydrogen peroxide, namely [Mo(O)(O_2_)_2_(H_2_O)_n_] (see Materials and Methods), in conjunction with **1c** and tetraphenylphosphonium bromide was employed to in-situ generate the catalyst. In these conditions, a 94% of conversion with high selectivity to sulfoxide (95%) and 40% *ee* to the (*R*)-sulfoxide was obtained [42]. A number of additional imidazolium-based zwitterionic dicarboxylic acids were also tested as chiral inductors in the enantioselective oxidation of methyl phenyl sulfide (Table 1). They are derived both from natural α-amino acids of general formula (*S*,*S*)-HL^R^ (R = Me, **1b**; CH_2_Ph, **1d**; ^i^Bu, **1e**, (*S*)-*sec*-Bu, **1f**) or non-natural α-amino acids, such as (*R*,*R*)-HL^iPr^ (**1c**’) and (*S*,*S*)-HL^tBu^ (**1g**) (Scheme 1). They were prepared by condensation of 2 equiv. of the corresponding amino acid with glyoxal and *p*-formaldehyde in water at 90 °C for one hour, following the procedures described in the literature [45,46]**.** Additionally, the new compounds (*S*,*S*)-HL^tBu^ (**1g**) and (*R*,*R*)-HL^iPr^ (**1c**’) were also straightforwardly obtained in an enantiopure form by the same procedure using the corresponding nonproteinogenic amino acids. Compounds **1g** and **1c**’ were characterized by IR, NMR (^1^H and ^13^C{^1^H}) and mass spectra (see Materials and Methods and Figures S1–S5 in Supplementary Materials). These compounds were employed to investigate the influence of (i) absence of chirality in the ligand (HL^H^, **1a**); (ii) size and branching of alkyl substituents (**1c**–**e**,**g**); (iii) an additional chiral center (**1f**); and (iv) a chiral center with opposed sense of chirality (**1c** vs **1c**’).

As expected, the [Mo(O)(O_2_)_2_(H_2_O)_n_]/HL^R^/[PPh_4_]Br system was effective for the sulfoxidation of methyl phenyl sulfide with conversions ranging from 67%, for **1d** (entry 5), to 93–95% for reagents **1a**–**c**, **1f** and **1g**. In all cases, reactions proceeded with chemoselectivity with nearly quantitative sulfoxide yields. The nature of the chiral inductor HL^R^ clearly controls enantioselectivity. The use of the achiral reagent **1a** gave the expected racemic mixture (entry 1). When reagents **1b**–**g** were employed, it was observed that an increase in the branching at the C_α_ atom of the R group of the ligand seemed to have a beneficial effect on the enantioselectivity. Specifically, reactions performed with chiral ligands with unbranched alkyl groups, such as **1b** and **1d** (entries 2 and 5, respectively), gave rise to low *ee* values of 2% and 5%, respectively. Conversely, the use of ligands with branched alkyl groups, such as **1c**, **1f** and **1g** (entries 3, 7 and 8, respectively), produced *ee* values higher than 30%. The highest *ee* was observed with the reagent **1f** (47% *ee*) in which the additional chiral center could have a positive effect in the enantioselectivity. Importantly, the reaction performed with (*R*,*R*)-HL^iPr^ (**1c**’) (entry 4) gave an *ee* result comparable to that of its (*S*,*S*)-enantiomer, **1c**, only with opposed sense of sulfoxide chirality. The adequate selection of the HL^R^ inductor chirality controls the production of the sulfoxide enantiomer. Finally, the activity of the system was tested with other sulfide substrates using compound **1f** as chiral inductor (entries 9–15). In general, good conversions and enantioselectivities close to 50% for the corresponding (*R*)-sulfoxide were found, with the exception of the sulfide Ph(HOCH_2_CH_2_)S, which showed lower values (29% sulfoxide yield and 43% *ee* for the (*S*) enantiomer, entry 13). Conversions obtained with **1f** were similar to those found with **1c** [42], but enantioselectivity values were slightly superior using **1f** than **1c** for the same substrates [42].

**Figure 1:**
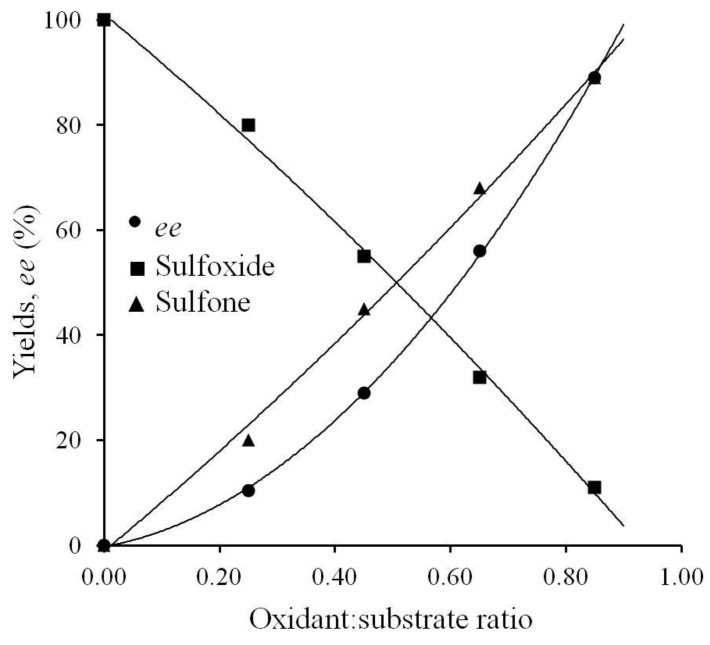
Kinetic resolution of racemic PhMeSO with catalyst [MoO(O_2_)_2_(H_2_O)_n_]/**1c**/[PPh_4_]Br (CHCl_3_, 0 °C, sulfoxide:Mo ratio of 100:2.5): sulfoxide and sulfone yields and the *ee* of the (*R*)-sulfoxide versus the oxidant:substrate ratio.

One equivalent of hydrogen peroxide per substrate was used in all experiments because formation of the corresponding sulfone was observed when two or more equivalents of the oxidant were employed [42,43]. As we previously communicated, the *ee* can be increased by kinetic resolution and the (*R*)-sulfoxide PhMeSO was obtained in 83% *ee* with a 1.6-fold excess of the oxidant [42]. To probe the kinetic resolution process in more detail, we performed the oxidation of racemic PhMeSO sulfoxide, under the same reaction conditions, varying the oxidant-to-substrate ratio (Figure 1). From the analysis of the variation of the enantiomeric excess with respect to the conversion of sulfoxide, it was possible to determinate a stereoselectivity factor *E* of 2.8 (*E* = kS’/kR’, see Supplementary Materials for details) [47]. Therefore, one may conclude that the enantiomeric excess of the sulfoxide can be controlled by adjusting the degree of conversion (at the expense of the sulfoxide yield).

### 2.2. Nature of the Molybdenum Catalyst and Origin of the Enantioselectivity

**Figure 2:**
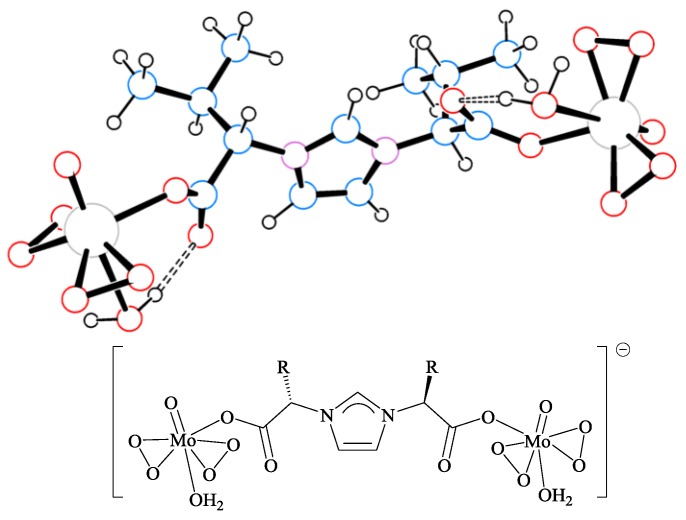
Optimized structure of the {[Mo(O)(O_2_)_2_(H_2_O)]_2_(μ-L^iPr^)}^−^ anion, **2c**, and proposed formulation of the Mo catalyst species.

With the purpose of gaining evidence about the nature of the molybdenum catalyst, the reaction of [Mo(O)(O_2_)_2_(H_2_O)_n_] with 2 equiv. of the sodium salt of (*S*,*S*)-HL^iPr^ was carried out. On the basis of Infrared (IR), Nuclear Magnetic Resonance (NMR) and Mass Spectrometry (MS) data (see Experimental and Supplementary Materials), the binuclear formulation Na{[Mo(O)(O_2_)_2_(H_2_O)]_2_(μ-L^iPr^)} was proposed for the isolated yellow powder. Further confirmation came from DFT calculations, which were carried out at the B3LYP level of theory for the anion {[Mo(O)(O_2_)_2_(H_2_O)]_2_(μ-L^iPr^)}^−^, **2c** (optimized structure shown in Figure 2). The computed IR spectrum of this anion fits well with the experimental one of complex Na{[Mo(O)(O_2_)_2_(H_2_O)]_2_(μ-L^iPr^)} (Figure S7, Supplementary Materials), thus supporting the proposed formulation. This allowed us to assign several IR absorptions of compound Na{[Mo(O)(O_2_)_2_(H_2_O)]_2_(μ-L^iPr^)}, as for instance the asymmetric and symmetric ν(COO) bands at 1611 and 1391 cm^−1^, respectively. This attribution gave a Δ(νCOO_asym_ − νCOO_sym_) value of ca. 220 cm^−1^, which is compatible with the monodentate κ^1^-O coordination of the carboxylate group observed in the optimized structure. Besides the carboxylate absorptions, the oxido group generates a characteristic ν(Mo=O) band at 962 cm^−1^, while the peroxide ligands display distinctive ν(OO), ν_as_[Mo(OO)] and ν_s_[Mo(OO)] absorptions at 861, 643 and 582 cm^−1^, respectively, in the expected ranges for this ligand [48].

In order to support the formulation of the Mo catalyst, the activity of the isolated complex Na{[Mo(O)(O_2_)_2_(H_2_O)]_2_(μ-L^iPr^)} was tested in the sulfoxidation reaction of methyl phenyl sulfide, under the optimized reaction conditions. The conversion (93%) and *ee* (42%) values achieved were completely similar to those observed when the catalytic species was in-situ formed [42], thus proving the nature of the catalyst as a binuclear {[Mo(O)(O_2_)_2_(H_2_O)]_2_(μ-L^R^)}^−^ oxidodiperoxidomolybdenum(VI) species.

Once the catalyst structure is known, the origin of the enantioselectivity was theoretically investigated. In principle, the simple κ^1^-*O*-carboxylate coordination of the chiral ligand (L^iPr^)^−^ can be in contradiction with the experimentally observed asymmetric process because chiral inductors are usually bi- or polydentate ligands. However, the analysis of the optimized structure {[Mo(O)(O_2_)_2_(H_2_O)]_2_(μ-L^iPr^)}^−^, **2c**, reveals two additional interactions. One is a hydrogen bond between the O–H from the water ligand and the noncoordinated oxygen atom of the carboxylate group of (L^iPr^)^−^ (explicitly shown in Figure 2, O–H···O distance of 2.75 Å). The second one is subtler and consists of a nonclassical hydrogen bond [49,50,51] between one C–H bond of the isopropyl group and one oxygen atom of one of the peroxido ligands (C–H···O distance of 2.51 Å). This interaction is also present in other optimized complexes {[Mo(O)(O_2_)_2_(H_2_O)]_2_(μ-L^R^)}^−^ (R = ^i^Bu, **2e**; ^sec^Bu, **2f**; and ^t^Bu, **2g**), while is weaker (for R = CH_2_Ph, **2d**) or absent in complexes containing R = H, **2a**, and Me, **2b**, substituents (see Table 2 and optimized structures in Figure S10 in Supplementary Materials). Compounds {[Mo(O)(O_2_)_2_(H_2_O)]_2_(μ-L^R^)}^−^ (R = ^i^Pr, **2c**; ^i^Bu, **2e**; ^sec^Bu, **2f**; and ^t^Bu, **2g**) display C–H···O distances within the range 2.50–2.66 Å and C–H···O angles higher than 160° (Table 2), which are typical parameters of nonclassical C–H···O hydrogen bonds (cut-off values of distances <2.8 Å and angles >90°) [52,53,54]. Interestingly, these compounds are those in which an asymmetric process is observed (inductors **1c**,**e**–**g** in Table 1), while for compounds without the C–H···O interaction, low or null activity is found (inductors **1a**,**b**,**d** in Table 1).

**Figure 3:**
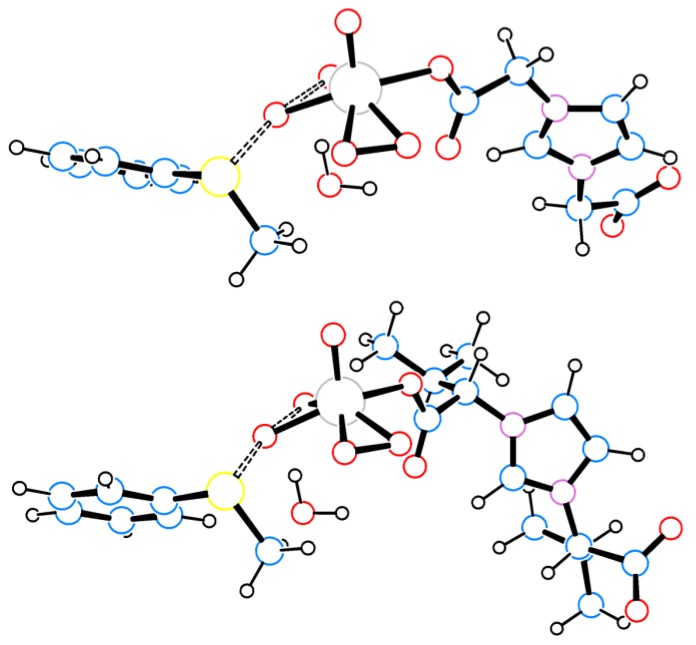
Calculated transition states for the oxido-transfer step from [Mo(O)(O_2_)_2_(H_2_O)(κ^1^-*O*-L^R^)]^−^ complexes to PhMeS (R = H, **TS_a1**, top; and iPr, **TS_c1**, bottom).

With the aim of confirming that these interactions are responsible of the asymmetry, we have selected the model complexes [Mo(O)(O_2_)_2_(H_2_O)(κ^1^-*O*-L^R^)]^−^ (R = H, **3a**, and ^i^Pr, **3c**), containing for simplicity only one molybdenum atom, and studied the step that controls the enantioselectivity. This is the oxido-transfer step, which follows a Sharpless-type outer-sphere concerted mechanism according to previous studies [43]. The oxygen atom transfer is produced by the nucleophilic attack of sulfide onto the peroxide ligand that cleaves the O–O bond with sulfoxide formation. This transition state (TS) reflects the interaction between the HOMO (Highest Occupied Molecular Orbital) of the sulfide substrate and the σ*(O–O) LUMO (Lowest Unoccupied Molecular Orbital) of peroxide and it is characterized by the approaching of sulfide reagent with associated elongation of the O–O linkage. Taking into account the presence of two peroxide ligands and two prochiral faces of the sulfide, four transition states have been located for the oxido-transfer. Figure 3 shows two of the calculated TSs for [Mo(O)(O_2_)_2_(H_2_O)(κ^1^-*O*-L^R^)]^−^ (R = H and iPr), while the other calculated TSs are shown in Figure S11 (Supplementary Materials). The four transition states optimized for the nonchiral [Mo(O)(O_2_)_2_(H_2_O)(κ^1^-*O*-L^H^)]^−^ species, **3a**, have the same Gibbs free energy (±0.2 kcal∙mol^−1^) with a barrier for the oxido-transfer step of ca. 35 kcal∙mol^−1^. The ∆∆G^≠^ is ca. 0 kcal∙mol^−1^, which is compatible with the formation of racemic sulfoxide using **1a** (entry 1, Table 1). By contrast, for chiral [Mo(O)(O_2_)_2_(H_2_O)(κ^1^-*O*-L^iPR^)]^−^ species, **3c**, there are two transition states, those that yield the *R* sulfoxide **TS_c1** and **TS_c4**, showing lower energies than **TS_c2** and **TS_c3** that afford the *S* sulfoxide. The calculated ∆∆G^≠^ of ca. 2 kcal∙mol^−1^ is well suited for the asymmetric process observed using **1c** (entry 3, Table 1).

## 3. Materials and Methods

### 3.1. General

Synthetic reactions were carried out under aerobic conditions. Chemicals were obtained from commercial sources and used as supplied, while solvents were appropriately purified using standard procedures. Infrared spectra were recorded on a Perkin-Elmer FT–IR Spectrum Two spectrophotometer (pressed KBr pellets). NMR spectra were recorded at the Centro de Investigaciones, Tecnología e Innovación (CITIUS) of the University of Sevilla by using Bruker AMX-300 or Avance III spectrometers with ^13^C{^1^H} and ^1^H shifts referenced to the residual solvent signals. All data are reported in ppm downfield from Si(CH_3_)_4_. The gas chromatograms (GC) were obtained using a Varian Chromatogram CP-3800 with nitrogen as the carrier gas. The chromatogram used a Varian automatic injector, model CP-8410, flame ionization detector (FID), and an Agilent column, model CP-7502. The HPLC chromatograms were performed on an Agilent 1260 Infinity instrument with a Chiralpak IA column at a flow rate of 1.0 mL/min with AcOEt/heptane = 6/4 (*v*/*v*) and using a UV detector at 254 nm. For Ph(HOCH_2_CH_2_)SO sulfoxide, a flow rate of 0.5 mL/min with heptane/^i^PrOH = 9/1 (*v*/*v*) was employed. The absolute configuration (reported in Table 1) was determined by comparing HPLC elution orders and the sign of the specific rotations with the literature data [14,15]. Polarimetry was carried out using a JASCO P-2000 Digital Polarimeter and the measurements were made at ca. 25 °C (concentration of ca. 10 mg/mL). High-resolution mass spectra (HRMS) were carried out by using a Q-Exactive Hybrid Quadrupole-Orbitrap Mass Spectrometer from Thermo Scientific at the CITIUS of the University of Sevilla.

### 3.2. Synthesis of Chiral Imidazolium-Based Zwitterionic Dicarboxylic Acids HL^R^

The syntheses of compounds (*S,S*)-HL^R^ (**1a**–**f**) have been previously described [45,46] and they were identified by comparison of their IR, NMR (^1^H and ^13^C{^1^H}) and mass spectra with those previously reported (see Figure S6, Supplementary materials).

*(R,R)-1-(1-carboxy-2-methylpropyl)-3-(1-carboxylate-2-methylpropyl)imidazolium*, (*R,R*)-HL^iPr^ (**1c**’). A solution of D-valine (10 g, 84 mmol) in water (25 mL) was reacted with glyoxal (4.80 mL, 40% *w*/*w* solution in water, 42 mmol) and formaldehyde (3.13 mL, 37% *w* solution in water, 42 mmol) at 95 °C for 2 h. Compound (*R,R*)-HLiPr, **1c**’, was obtained by removing the solvent under reduced pressure. Recrystallization from water yields 5.18 g (46%) of the product as light-brown solid. IR (KBr, cm^−1^): 3464 (br), 3166 (w), 3114 (m), 3046 (m), 2970 (s), 2935 (w), 2878 (m), 1686 (vs,br), 1548 (s), 1473 (m), 1392 (m), 1375 (m), 1344 (w), 1295 (w), 1265 (m), 1162 (s), 1120 (m), 1096 (m), 1015 (w), 977 (w), 912 (w), 871 (w), 838 (w), 760 (w), 712 (w), 652 (w). ^1^H NMR (300 MHz, D_2_O): *δ* 0.91, 1.00 (d, ^3^*J*_HH_ = 6.6 Hz, 6H, CH(C*H*_3_)_2_), 2.55 (m, 2H, C*H*(CH_3_)_2_), 4.84 (d, ^3^*J*_HH_ = 7.8 Hz, 2H, C*H*^i^Pr), 7.68 (s, 2H, C^4^*H*/C^5^*H*), 9.13 (s, 1H, C^2^*H*). ^13^C{^1^H} NMR (75 MHz, D_2_O): *δ* 17.3, 18.4 (s, CH(*C*H_3_)_2_), 31.2 (s, *C*H(CH_3_)_2_), 69.8 (s, *C*H^i^Pr), 122.3 (s, *C*^4^H/*C*^5^H), 136.2 (s, *C*^2^H), 172.3 (s, *C*O). ${\left[\alpha \right]}_{D}^{25}=-106.5$ (H_2_O). HRMS for C_13_H_20_N_2_O_4_: [M + 1]^+^ requires *m*/*z* 269.15, found *m*/*z* 269.1492.

*(S,S)-1-(1-carboxy-2,2-dimethylpropyl)-3-(1-carboxylate-2,2-dimethylpropyl) imidazolium*, (*S,S*)-HL^tBu^ (**1g**). A solution of L-*tert*-leucine (2 g, 15 mmol) in water (20 mL) was reacted with glyoxal (866 μL, 40% *w* solution in water, 8 mmol) and formaldehyde (566 μL, 37% *w* solution in water, 8 mmol) at 95 °C for 4 h. Compound (*S,S*)-HL^tBu^, **1g**, was obtained by removing the solvent under reduced pressure. Recrystallisation from water yields 1.83 g (82%) of the product as light-brown solid. IR (KBr, cm^−1^): 3452 (br), 3187 (m), 3160 (m), 3108 (m), 3038 (m), 2965 (s), 2915 (w), 2878 (w), 1686 (vs,br), 1553 (s), 1482 (s), 1447 (w), 1403 (m), 1375 (s), 1369 (m), 1353 (m), 1315 (m), 1268 (m), 1215 (m), 1159 (s), 1101 (m), 1051 (m), 1029 (w), 938 (m), 892 (m), 855 (m), 820 (w), 801 (w), 789 (m), 769 (m), 731 (s), 699 (m), 681 (m), 657 (m), 643 (m). ^1^H NMR (300 MHz, CD_3_OD): *δ* 1.10 (s, 18H, C(C*H*_3_)_3_), 4.87 (s, 2H, C*H*^t^Bu), 7.75 (d, ^4^*J*_HH_ = 1.5 Hz, 2H, C^4^*H*/C^5^*H*), 9.49 (s, 1H, C^2^*H*). ^13^C{^1^H} NMR (75 MHz, CD_3_OD): *δ* 26.0 (s, C(*C*H_3_)_3_), 34.7 (s, *C*(CH_3_)_3_), 72.6 (s, *C*H^t^Bu), 122.3 (s, *C*^4^H/*C*^5^H), 137.3 (s, *C*^2^H), 169.6 (s, *C*O). ${\left[\alpha \right]}_{D}^{25}=+144.4$ (H_2_O). HRMS for C_15_H_24_N_2_O_4_: [M + 1]^+^ requires *m*/*z* 297.18, found *m*/*z* 297.1804.

### 3.3. Preparation and Titration of [Mo(O)(O_2_)_2_(H_2_O)_n_] Solution

Solutions of the aqua complex of oxidodiperoxidomolybdenum in aqueous hydrogen peroxide were prepared as previously described [55]. For the purpose of simplicity the solution is referred to in this work simply as aqueous [Mo(O)(O_2_)_2_(H_2_O)_n_].

The resulting aqueous solution of molybdenum complex has an excess of hydrogen peroxide. The addition of the 0.025 mmol of molybdenum species in the catalytic essays includes a supplementary amount of oxidant. In order to avoid the formation of sulfone product, one equivalent of 30% hydrogen peroxide per sulfide substrate should be used. Thus, freshly prepared [Mo(O)(O_2_)_2_(H_2_O)_n_] 0.25 M solutions were employed, which were conveniently titrated before each catalytic test. The titration was carried out as follows. To 10 mL of [Mo(O)(O_2_)_2_(H_2_O)_n_] solution was added H_2_SO_4_ 6M (10 mL) and the mixture diluted with water (25 mL). This solution was titrated with KMnO_4_ ca. 0.2 M (previously standardized with Na_2_C_2_O_4_). The mean of five titrations afforded a typical value of ca. 0.2 mmol of hydrogen peroxide per 100 μL of solution. On this basis, the exact amount of hydrogen peroxide 30% employed in the catalytic test can be easily calculated.

### 3.4. Synthesis of Complex Na{[Mo(O)(O_2_)_2_(H_2_O)]_2_(μ-L^iPr^)}

Over a solution of (*S*,*S*)-HL^iPr^ (0.504 g, 1.88 mmol) in water (15 mL) was added dropwise a solution of NaHCO_3_ (0.158 g, 1.88 mmol) in water (5 mL) and the mixture was stirred at room temperature until the evolution of CO_2_ ceased (5–10 min). Over this solution was added [Mo(O)(O_2_)_2_(H_2_O)_n_] (15.03 mL, 0.25 M aqueous solution, 3.76 mmol) and the mixture was stirred at room temperature for 1 h. The resulting solution was evaporated to dryness affording a yellow powder identified as Na{[Mo(O)(O_2_)_2_(H_2_O)]_2_(μ-L^iPr^)} (1.216 g, 96%). IR (KBr, cm^−1^): 3440 (br vs), 3136 (m), 2966 (s), 2935 (m), 2877 (m), 1611 (vs), 1563 (m), 1548 (m), 1468 (m), 1423 (m), 1391 (s), 1344 (m), 1258 (m), 1234 (w), 1155 (s), 1120 (m), 1025 (w), 962 (s), 861 (s), 750 (m), 712 (w), 643 (m), 582 (m), 537 (m). ^1^H NMR (D_2_O, 300 MHz): δ 0.80 (br d, 6H, 2CH(C*H*_3_)_2_), 0.91 (br d, 6H, 2×CH(C*H*_3_)_2_), 2.44 (br m, 2H, 2×C*H*(CH_3_)_2_), 4.58 (br m, 2H, 2×C*H*^i^Pr), 7.53 (s, 2H, 2×C*H*, H^4^/H^5^), 8.92 (s, 1H, C*H*, H^2^). ^13^C{^1^H} NMR (D_2_O, 75 MHz): δ 17.5 (s, 2×CH(*C*H_3_)_2_), 18.6 (s, 2×CH(*C*H_3_)_2_), 31.1 (s, 2×*C*H(CH_3_)_2_), 71.4 (s, 2×*C*H^i^Pr), 122.0 (s, 2×*C*H, C^4^/C^5^), 135.8 (s, *C*H), 173.6 (s, 2×*C*OO). Electrospray ionization-MS: positive mode, found *m*/*z* 269.15 (for HL^iPr^ + 1, C_13_H_20_N_2_O_4_, 268.14) and 291.13 (for NaL^iPr^ + 1, NaC_13_H_19_N_2_O_4_, 290.12). ESI-MS: negative mode, found *m*/*z* 267.13 (for HL^iPr^-1, C_13_H_20_N_2_O_4_, 268.14), 445.01 (for MoO_5_L^iPr^-1, C_13_H_20_MoN_2_O_9_, 446.02).

### 3.5. General Procedure for Enantioselective Mo-Catalyzed Oxidation of Sulfides in the Presence of HL^R^

The reactor (a 50 mL vial equipped with a Young valve and containing a stirrer flea) was charged with [Mo(O)(O_2_)_2_(H_2_O)_n_] (100 μL, 0.25 M aqueous solution, 0.025 mmol), HL^R^ (0.0125 mmol), [PPh_4_]Br as specified (typically 0.05 mmol), the reaction solvent (1 mL), the oxidant (30% aqueous H_2_O_2_; 1 mmol per sulfide substrate, see details above) and the sulfide substrate (1 mmol), in the aforementioned order. The reactor was sealed and maintained at the working temperature, with constant stirring (600 rpm) in a thermostatted bath for the duration of the reaction. Upon completion, the reaction mixture was treated with diethyl ether (10 mL) and then filtered with 0.45 μm nylon syringe filter. The resulting solution was analyzed by GC (by adding 50 μL of dodecane as the internal standard). Afterwards the solution was evaporated to dryness by using a rotavap. The resulting residue was then analyzed by HPLC (by adding 20 mL of ethyl acetate).

### 3.6. Computational Details

The electronic structure and geometries of the model compounds [Mo(O)(O_2_)_2_(H_2_O)]_2_(μ-L^R^)]^−^ (R = H, **2a**; Me, **2b**; ^i^Pr, **2c**; CH_2_Ph, **2d**; ^i^Bu, **2e**; ^sec^Bu, **2f**; and ^t^Bu, **2g**) and [Mo(O)(O_2_)_2_(H_2_O)(κ^1^-*O*-L^R^)]^−^ (R = H, **3a**, and ^i^Pr, **3c**) were computed using density functional theory at the B3LYP level [56,57]. The Mo atom was described with the LANL2DZ basis set [58,59] while the 6-31G(d,p) basis set was used for the C, N, O, S and H atoms. The transition states of the interaction of PhMeS with **3a** and **3c**, namely **TSa1**–**4** and **TSc1**–**4**, were located at the same level of theory. Geometries of all model complexes were optimized without symmetry constraints. Frequency calculations were carried out at the same level of theory to identify all of the stationary points as transition states (one imaginary frequency) or as minima (zero imaginary frequencies) and to provide the thermal correction to free energies at 298.15 K and 1 atm. The DFT calculations were performed using the Gaussian 09 suite of programs [60]. Coordinates of the optimized compounds are collected in Table S4 (Supplementary Materials).

## 4. Conclusions

A simple process for the enantioselective Mo-catalyzed sulfoxidation with aqueous hydrogen peroxide, by using imidazolium-based dicarboxylate compounds HL^R^, **1b**–**g**, as chiral inductors, has been developed. The advantages of this system are: (i) better reaction times (1 h); (ii) commercial and cheap molybdenum starting material, MoO_3_; and (iii) straightforward synthesis of the (*S*,*S*)- or (*R*,*R*)-HL^R^ inductors, in comparison with the elaborated chiral ligands reported in the bibliography. By combination of spectroscopic data and DFT calculations, the binuclear anion {[Mo(O)(O_2_)_2_(H_2_O)]_2_(μ-L^R^)}^−^, **2**, has been proposed as the chiral catalytic species. A nonclassical hydrogen bond between one C–H bond of the alkyl R group and one oxygen atom of one of the peroxido ligands controls the enantioselectivity of the sulfoxidation. This subtle interaction is only present in optimized complexes **2c**,**e**,**f**,**g**, those that showed an acceptable *ee* value. This has been additionally demonstrated by analysing the transition states of the oxido-transfer of model complexes [Mo(O)(O_2_)_2_(H_2_O)(κ^1^-*O*-L^R^)]^−^ (R = H, **3a**, and ^i^Pr, **3c**) to PhMeS sulfide.

## Figures and Tables

**Table 1:** Enantioselective oxidation of different sulfides with the system [MoO(O_2_)_2_(H_2_O)_n_]/H_2_O_2_/HL^R^/[PPh_4_]Br ^a^.

Entry	HLR	Sulfide	Conversion (%) ^b^	Selectivity to Sulfoxide (%) ^b^	Selectivity to Sulfone (%) ^b^	Sulfoxide Yield (%)	Sulfoxide *ee* (%) and Configuration ^c^
1	HL^H^, **1a**	PhMeS	93	95	5	88	Racemic
2	(*S*,*S*)-HL^Me^, **1b**	PhMeS	93	95	5	88	2 (*R*)
3	(*S*,*S*)-HL*^i^*^Pr^, **1c**	PhMeS	94	95	5	89	40 (*R*)
4	(*R*,*R*)-HL*^i^*^Pr^, **1c**’	PhMeS	95	95	5	90	42 (*S*)
5	(*S*,*S*)-HL^CH2Ph^, **1d**	PhMeS	67	100	0	67	5 (*R*)
6	(*S*,*S*)-HL*^i^*^Bu^, **1e**	PhMeS	88	96	4	85	14 (*R*)
7	(*S*,*S*)-HL*^sec-^*^Bu^, **1f**	PhMeS	95	95	5	90	47 (*R*)
8	(*S*,*S*)-HL*^t^*^Bu^, **1g**	PhMeS	92	96	4	88	32 (*R*)
9	(*S*,*S*)-HL*^sec-^*^Bu^, **1f**	(*p*-Me-C_6_H_4_)MeS	90	91	9	82	55 (*R*)
10	(*S*,*S*)-HL*^sec-^*^Bu^, **1f**	(*p*-Cl-C_6_H_4_)MeS	89	96	4	85	44 (*R*)
11	(*S*,*S*)-HL*^sec-^*^Bu^, **1f**	(*p*-Br-C_6_H_4_)MeS	91	87	13	79	51 (*R*)
12	(*S*,*S*)-HL*^sec-^*^Bu^, **1f**	Ph(PhCH_2_)S	90	64	36	58	53 (*R*)
13	(*S*,*S*)-HL*^sec-^*^Bu^, **1f**	Ph(HOCH_2_CH_2_)S	81	36	0	29	43 (*S*)

^a^ Reaction conditions: catalyst [MoO(O_2_)_2_(H_2_O)_n_] 0.025 mmol, HL^R^ 0.0125 mmol, [PPh_4_]Br 0.05 mmol, sulfide 1.0 mmol, solvent: Cl_3_CH 1.0 mL, oxidant: H_2_O_2_ (30% aq.), oxidant:sulfide ratio 1:1, 1 h, T = 0 °C. ^b^ Determined by Gas Chromatography (50 μL of dodecane as the internal standard). ^c^ Determined by High-Performance Liquid Chromatography (HPLC, see details in Supplementary Materials).

**Table 2:** Selected structural data for classical and nonclassical hydrogen bonds in optimized structures {[Mo(O)(O_2_)_2_(H_2_O)]_2_(μ-L^R^)}^−^ **2**.

R (μ–L^R^)^−^		Distances, Å		Angles, °	
		C–H···O	O–H···O	C–H···O	O–H···O
H	**2a**	-	1.814, 1.822	-	158
Me	**2b**	>4	1.814, 1.831	-	158, 159
^i^Pr	**2c**	2.509, 2.521	1.807, 1.817	168	159, 160
CH_2_Ph	**2d**	2.352 (C–H_arom._), 3.570	1.803, 1.815	138 (C–H_arom._), 111	160
^i^Bu	**2e**	2.552, 2.558	1.796, 1.811	170, 171	160
^sec^Bu	**2f**	2.621, 2.656	1.793, 1.803	160	160, 161
^t^Bu	**2g**	2.509, 2.521	1.807, 1.817	166	160

## Supplementary Materials

The following are available online, Figures S1–S6: NMR and MS spectra of **1** compounds, Figure S7: calculated IR spectrum of **2c**, Figures S7 and S8: determination of the stereoselectivity factor, Figures S10 and S11: optimized structures of transition states and compounds **2**, Figure S12: selected chiral HPLC diagrams of optical active sulfoxides, Table S1: energies of the transition states for the oxido-transfer, and Table S2: Coordinates of the optimized structures.
