# Supplementary material for: Molybdenum-Catalyzed Enantioselective Sulfoxidation Controlled by a Nonclassical Hydrogen Bond between Coordinated Chiral Imidazolium-Based Dicarboxylate and Peroxido Ligands
Source: Molecules. 2018 Jun 30;23(7):1595. doi: 10.3390/molecules23071595 (PMC6100257; doi:10.3390/molecules23071595)
Supplement: Supplementary file 1 [file molecules-23-01595-s001.pdf]

Supplementary Materials

Molybdenum-catalyzed enantioselective sulfoxidation controlled by a non-classical hydrogen bond between coordinated chiral imidazolium-based dicarboxylate and peroxido ligands: experimental and DFT studies

Carlos J. Carrasco, Francisco Montilla* and Agustín Galindo*

- **Figs. S1 and S2.** ^{1}H and $^{13}C\{^{1}H\}$ NMR spectra of **1c'**.
- **Figs. S3 and S4.** ^{1}H and $^{13}C\{^{1}H\}$ NMR spectra of **1g**.
- **Fig. S5.** Mass spectra of compounds **1c'** and **1g**.
- **Fig. S6.** NMR (^{1}H and $^{13}C\{^{1}H\}$) and mass spectra of known compounds **1a**-**f**.
- **Fig. S7.** Comparison of the IR spectrum of complex $Na\{[Mo(O)(O_2)_2(H_2O)]_2(\mu\text{-}L^{iPr})\}$ (experimental) with the calculated IR spectrum of the $\{[Mo(O)(O_2)_2(H_2O)]_2(\mu\text{-}L^{iPr})\}^-$ anion, **2c**.
- **Figs. S8 and S9.** Determination of the stereoselectivity factor ($E = k_S'/k_R'$) of kinetic resolution.
- **Fig. S10.** Optimized structures of compounds $\{[Mo(O)(O_2)_2(H_2O)]_2(\mu\text{-}L^{R})\}^-$ **2**.
- **Fig. S11.** Optimized structures of the transition states for the oxido-transfer to PhMeS from $[Mo(O)(O_2)_2(H_2O)(\kappa^1\text{-}O\text{-}L^R)]^-$ (R = H, top; and ^{i}Pr, bottom) complexes.
- **Fig. S12.** Selected chiral HPLC diagrams of optical active sulfoxides with different *ee* (entries 9-13 of Table 1) and comparison with racemic mixtures.
- **Table S1.** Calculated energies (Hartree) of the transition states for the oxido-transfer to PhMeS from $[Mo(O)(O_2)_2(H_2O)(\kappa^1\text{-}O\text{-}L^R)]^-$ (R = H, ^{i}Pr) complexes.
- **Table S2.** Coordinates of the optimized structures.

Fig. S1. ^{1}H NMR spectrum of **1c'**.

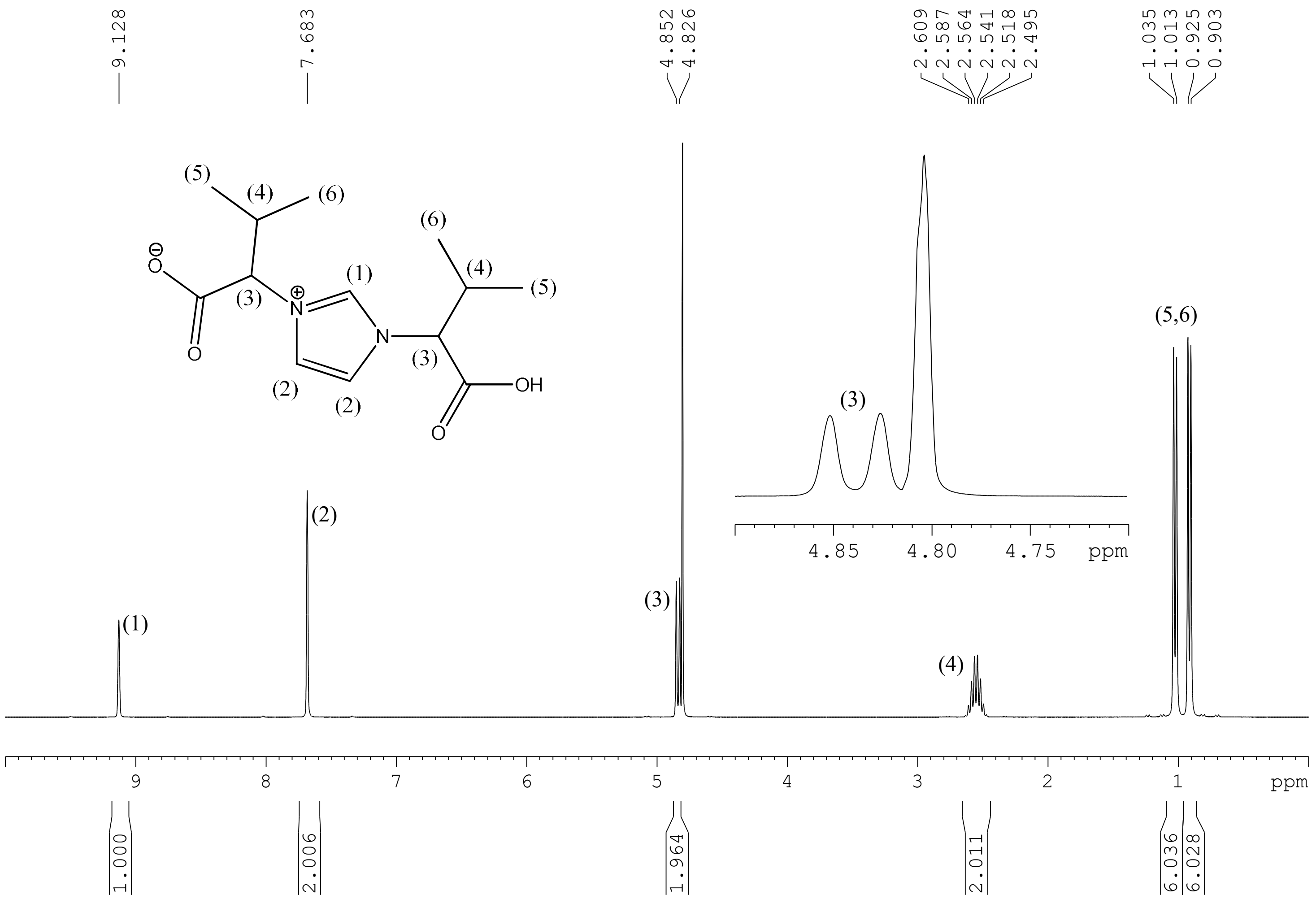

Fig. S2. $^{13}C\{^{1}H\}$ NMR spectrum of **1c'**.

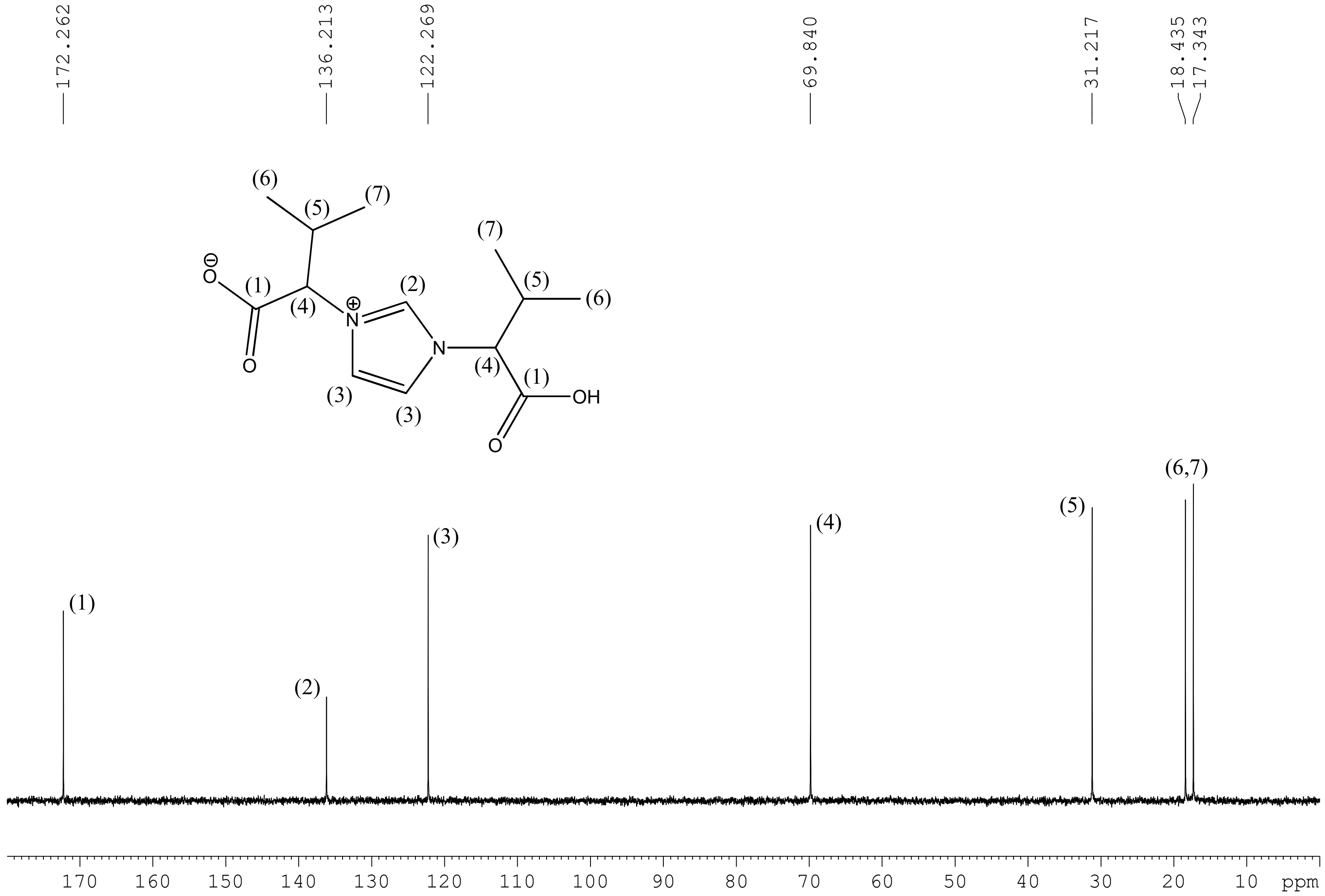

Fig. S3. ^{1}H NMR spectrum of **1g**.

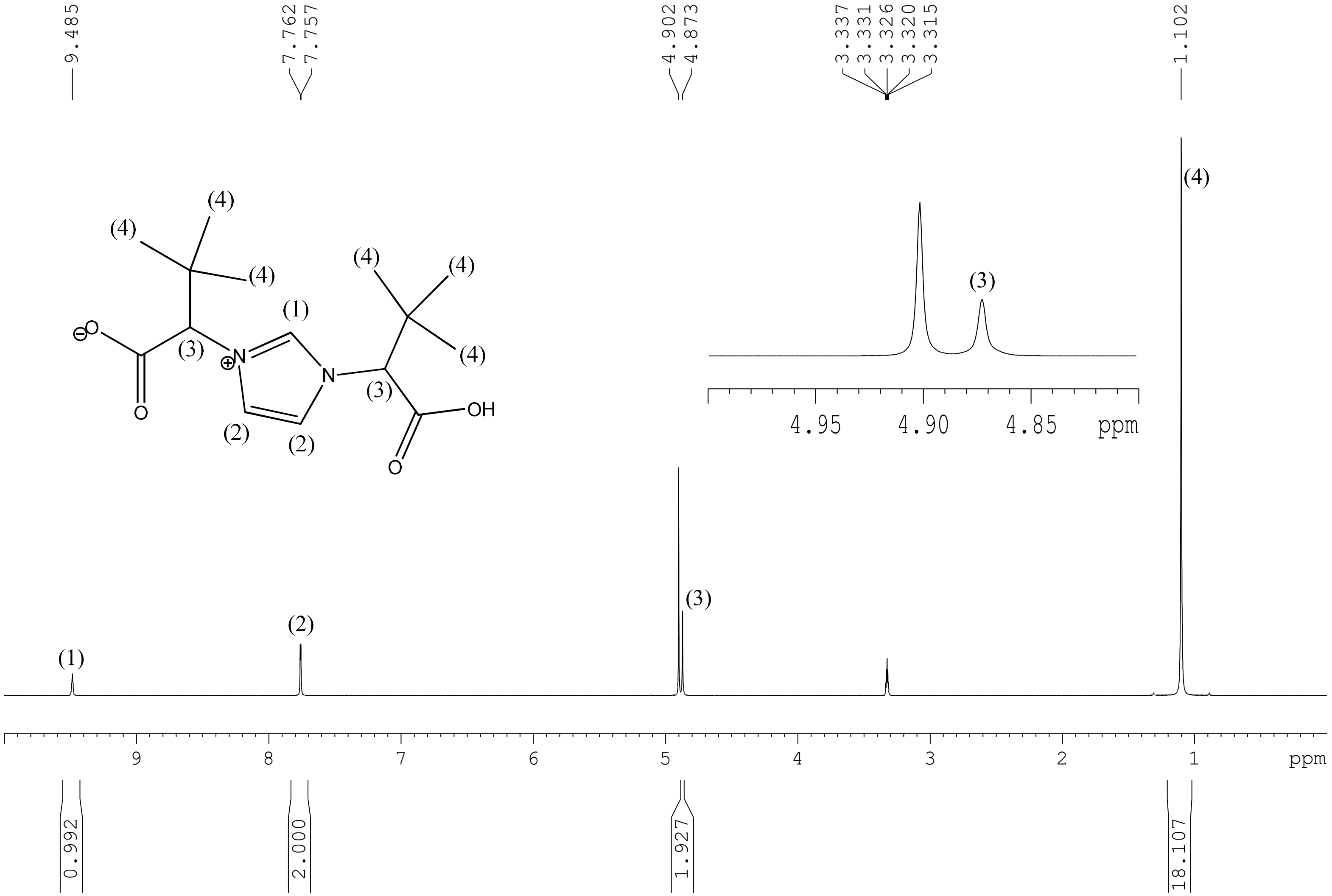

Fig. S4. $^{13}C\{^1H\}$ NMR spectrum of **1g**.

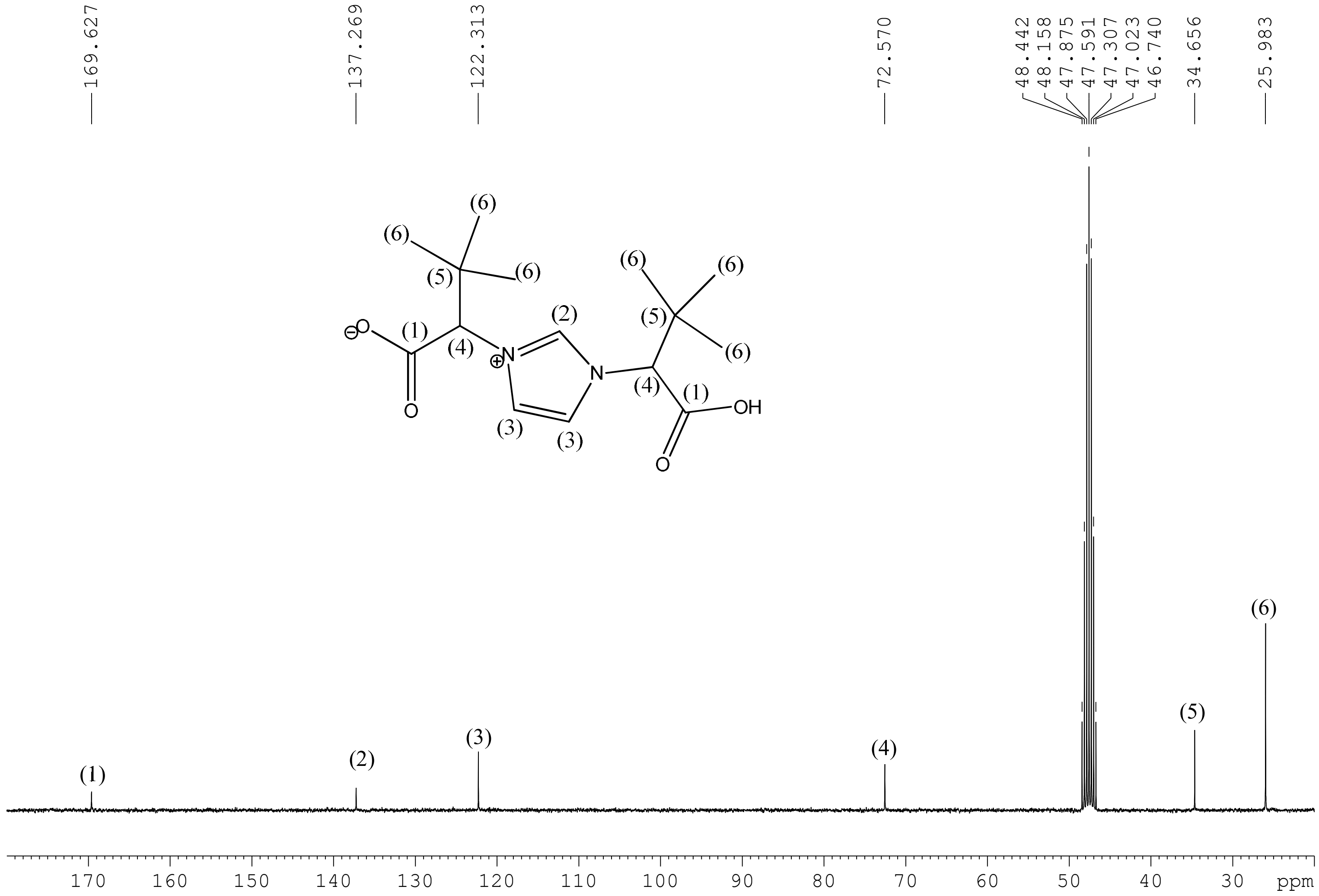

Fig. S5. Mass spectra of compounds **1c'** and **1g**.

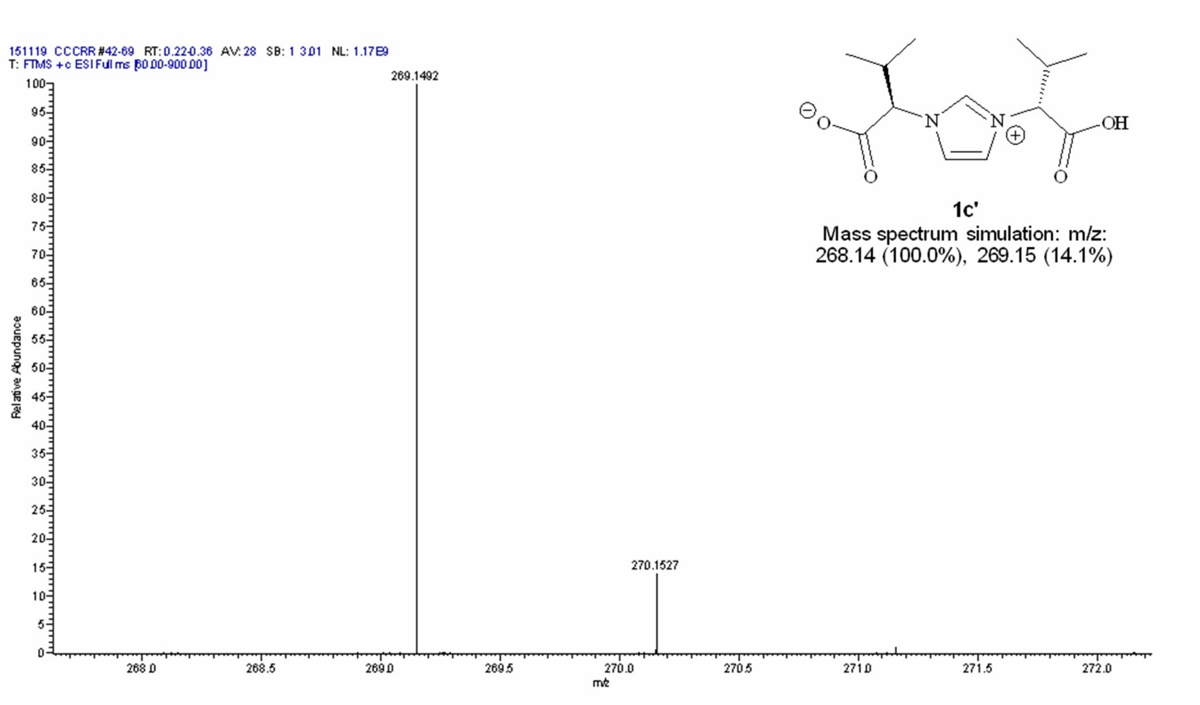

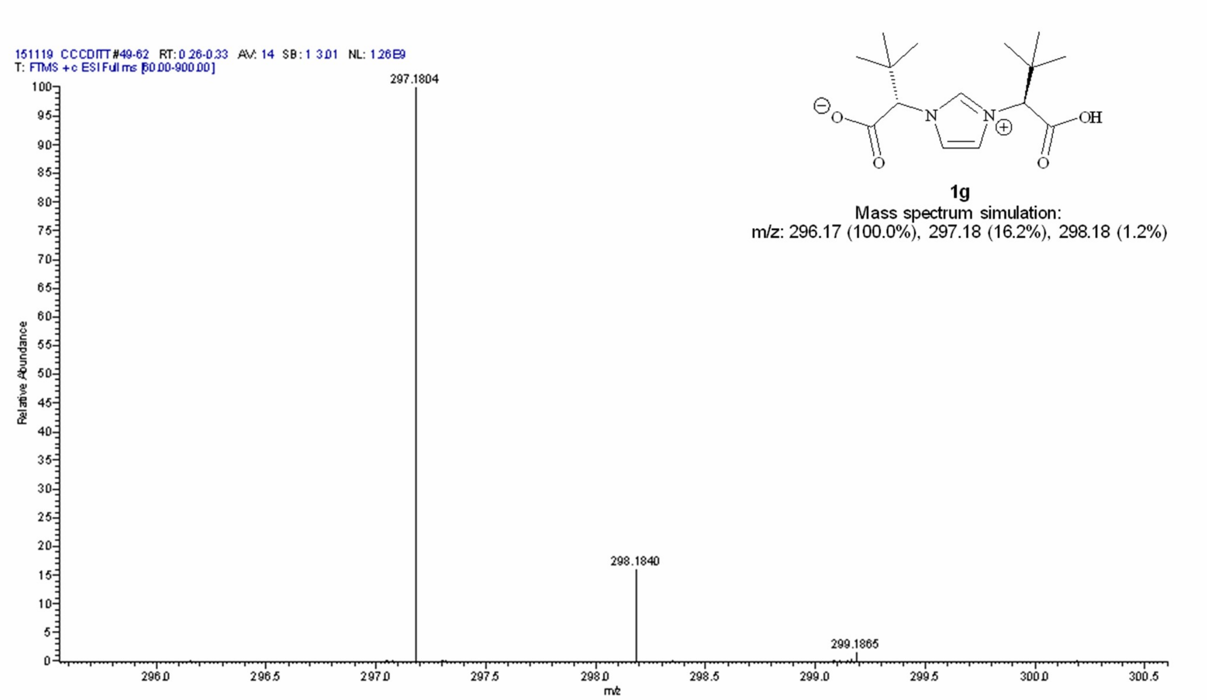

Fig. S6. NMR (^{1}H and ^{13}C{^{1}H}) and mass spectra of known compounds **1a-f**.

^{1}H NMR spectrum of **1a**.

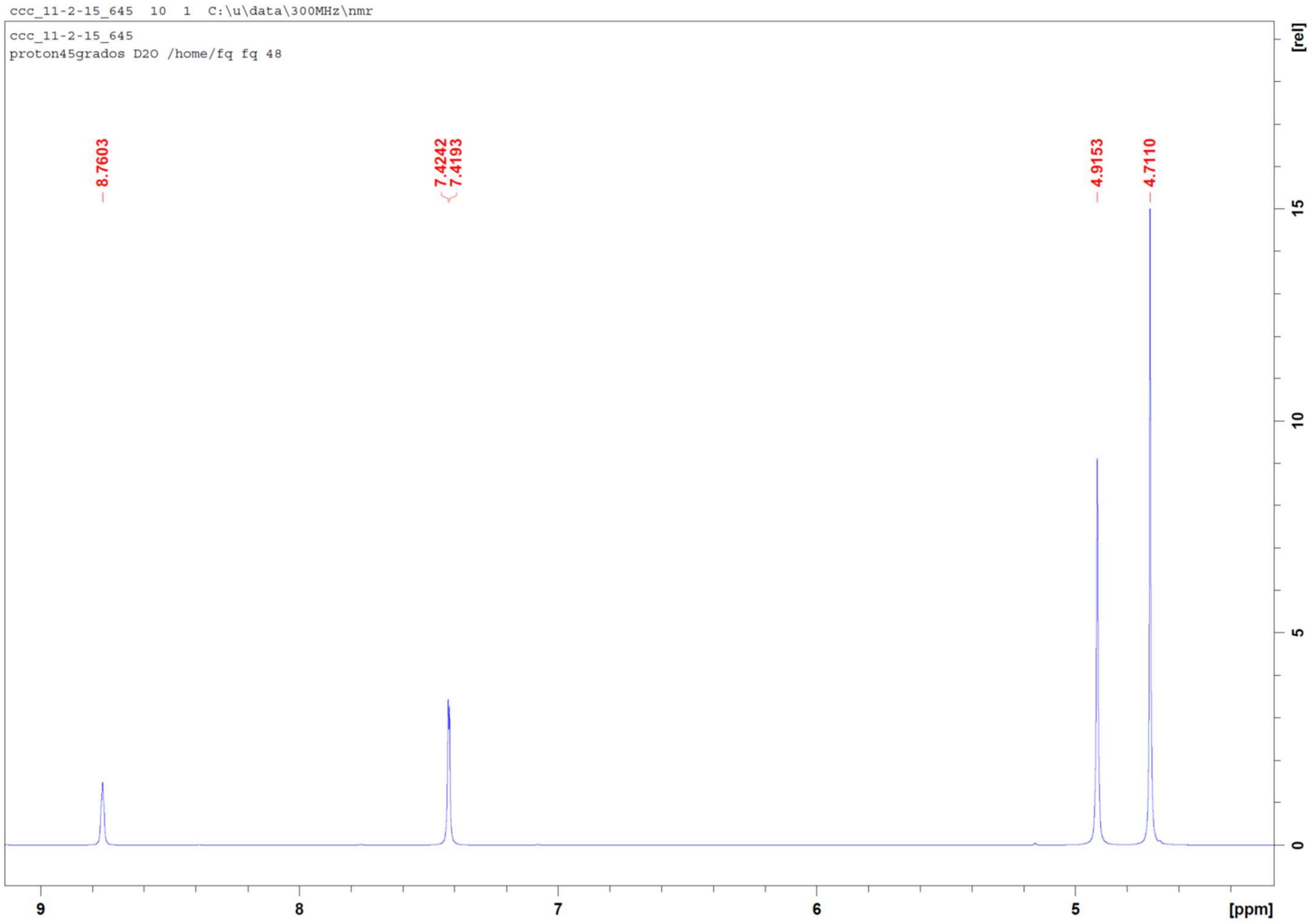

$^{13}C\{^1H\}$ NMR spectrum of **1a**.

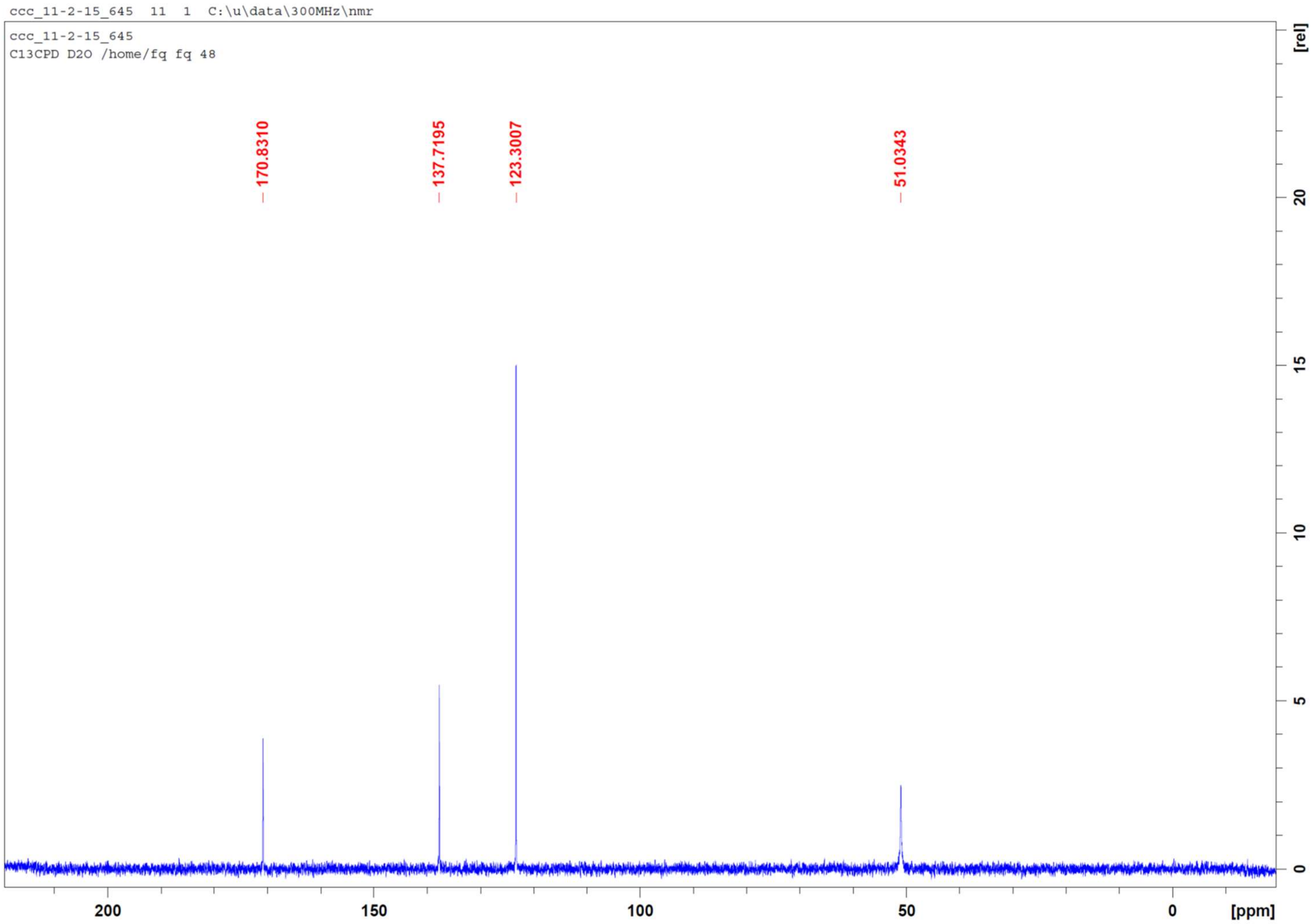

MS spectrum of **1a**.

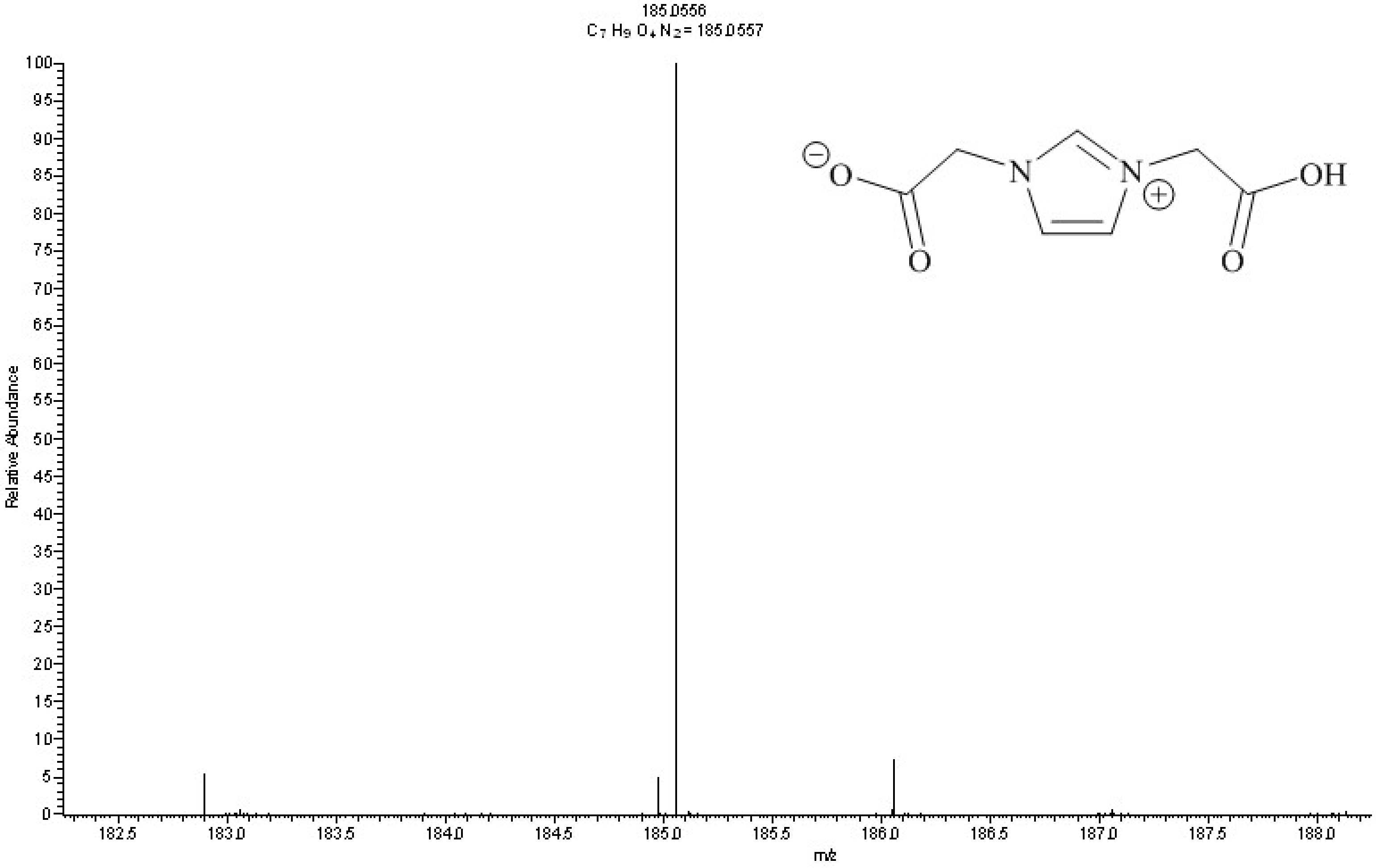

^{1}H NMR spectrum of **1b**.

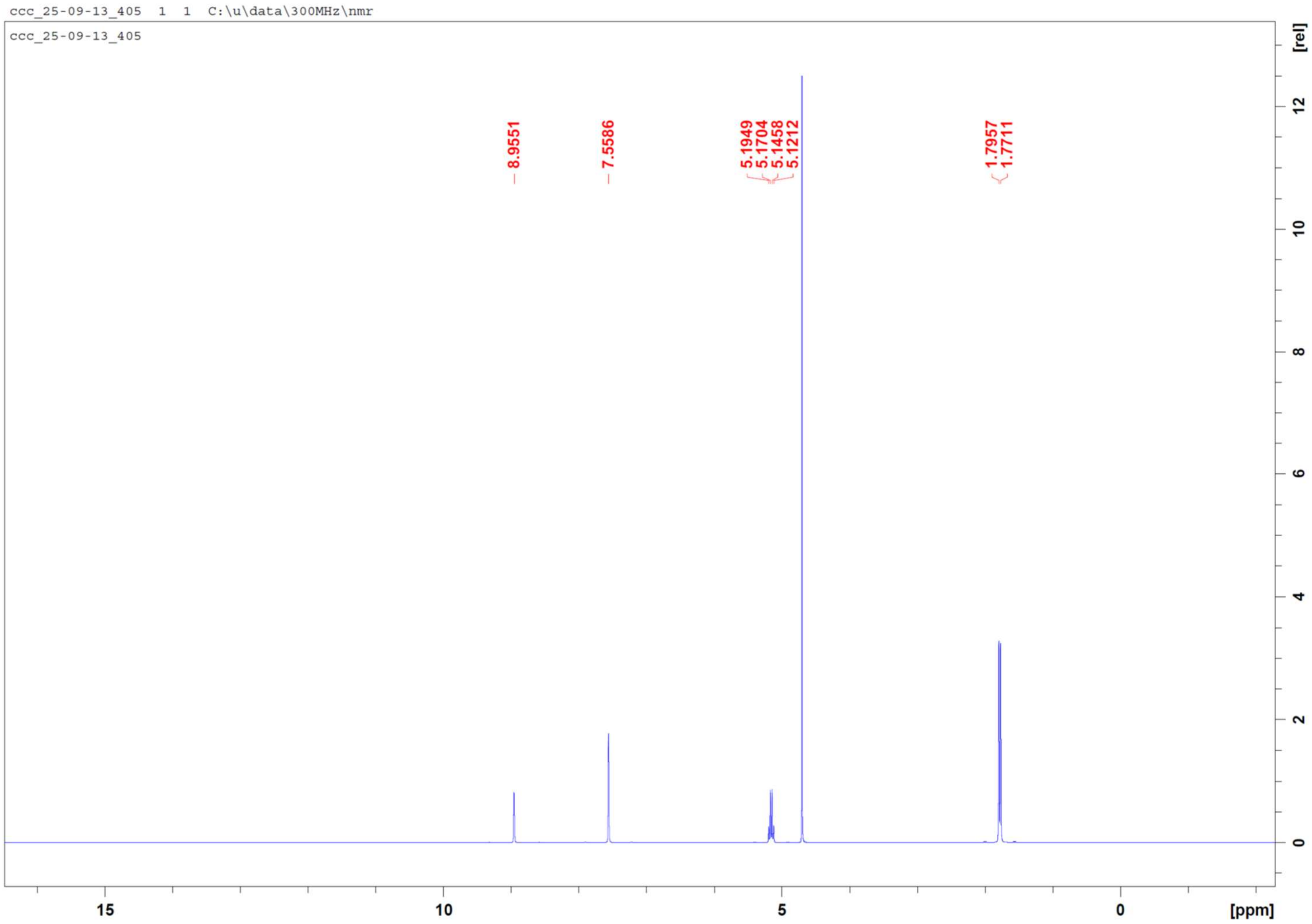

$^{13}C\{^{1}H\}$ NMR spectrum of **1b**.

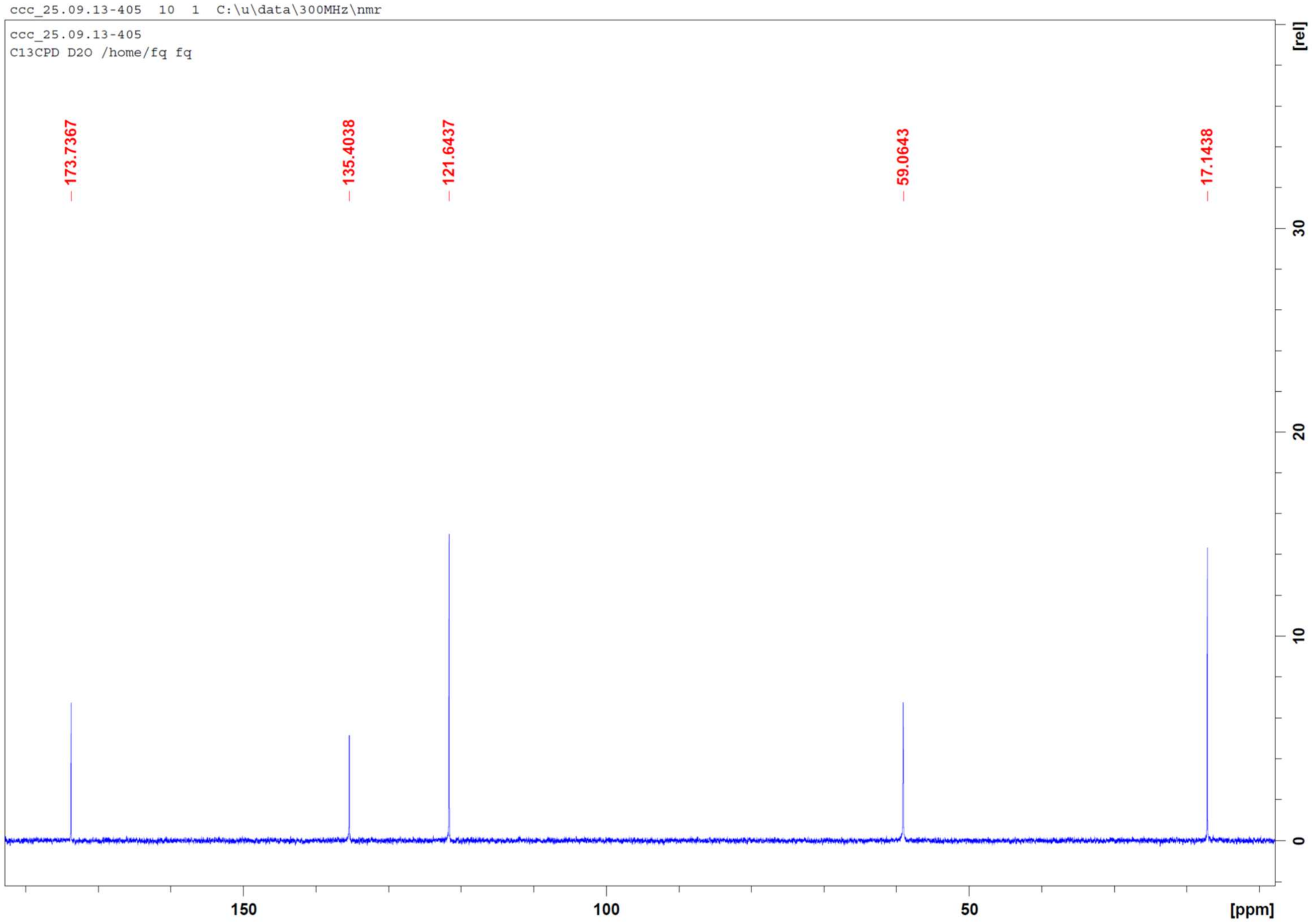

MS spectrum of **1b**.

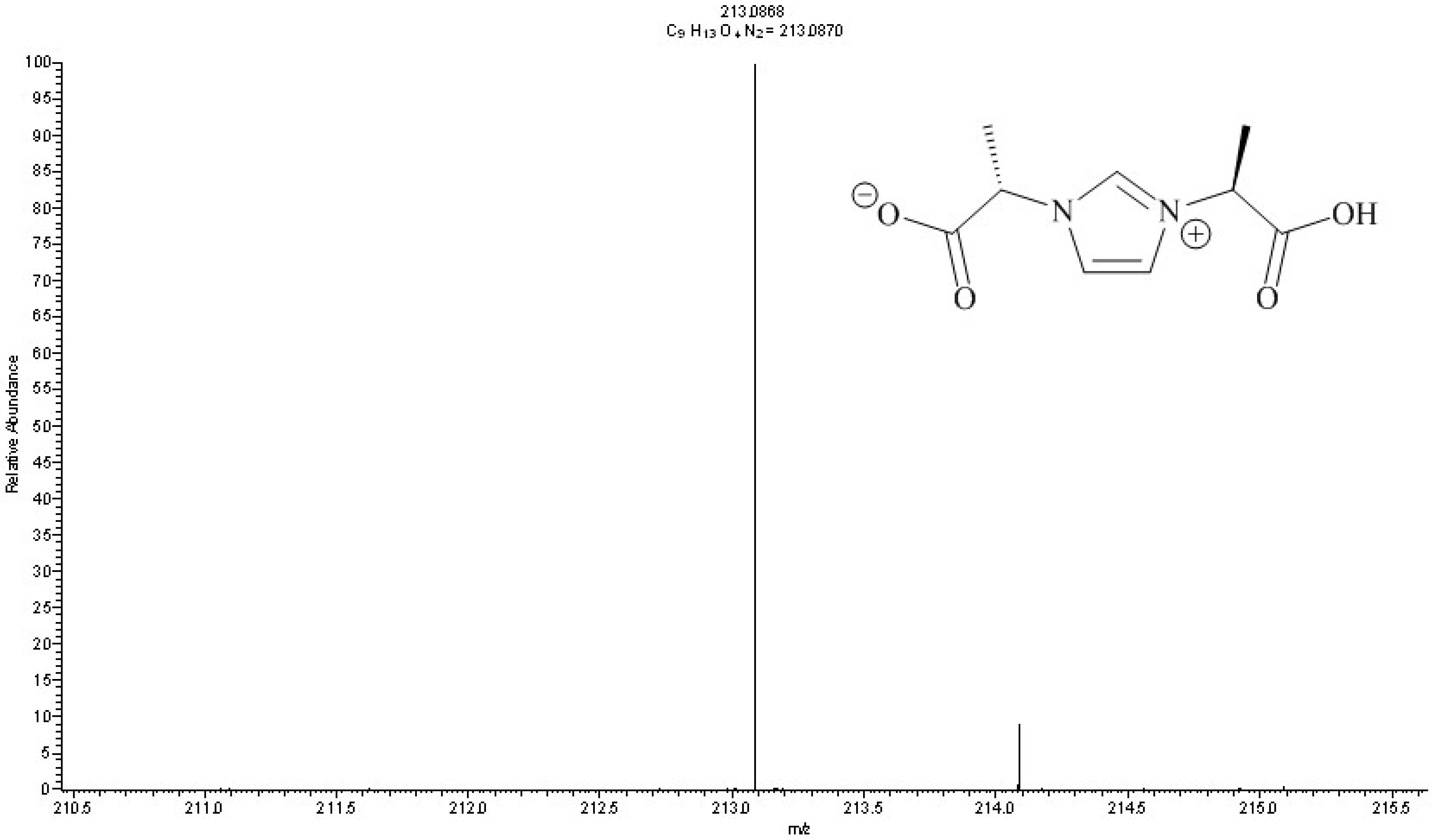

^{1}H NMR spectrum of **1c**.

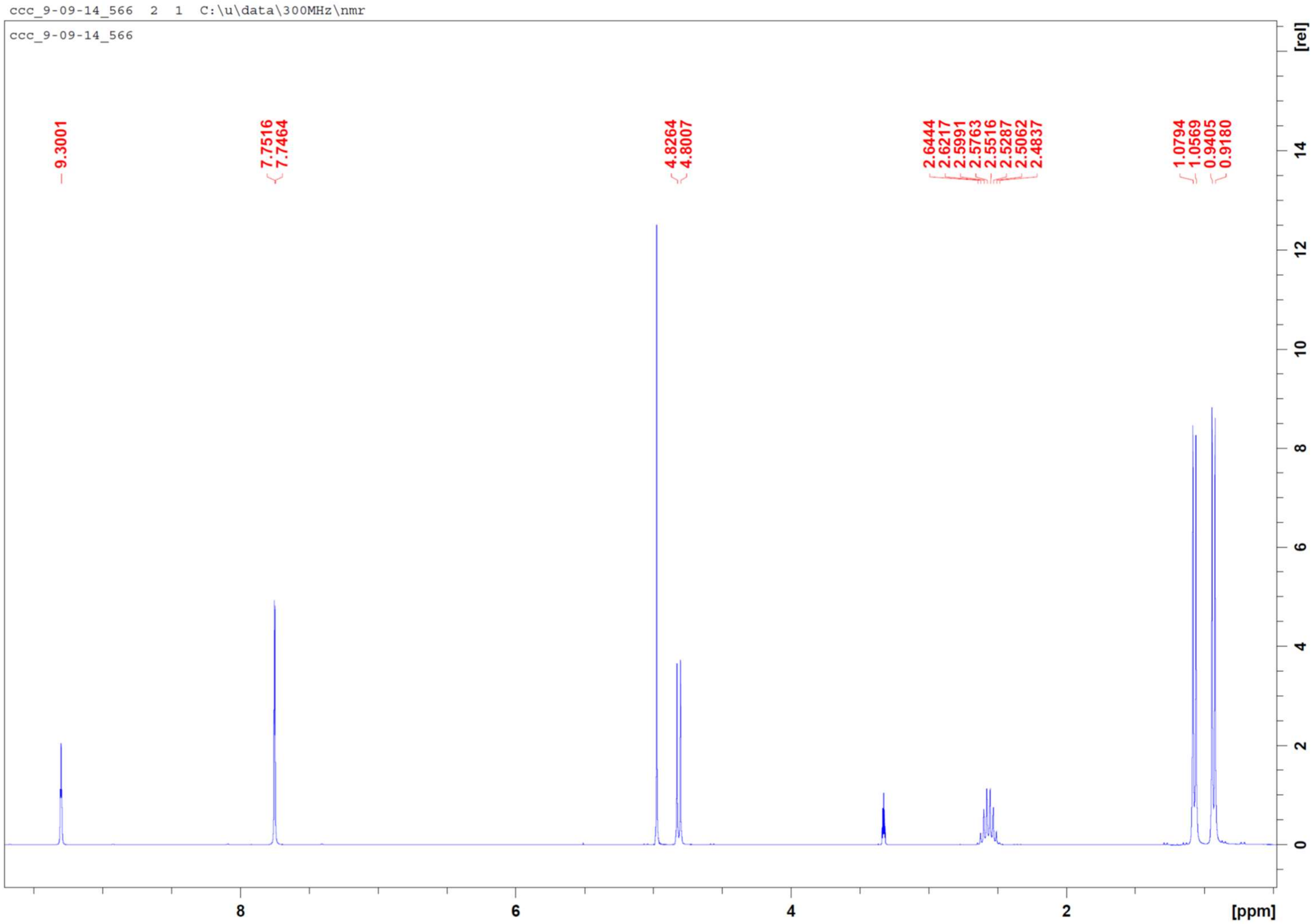

$^{13}C\{^1H\}$ NMR spectrum of **1c**.

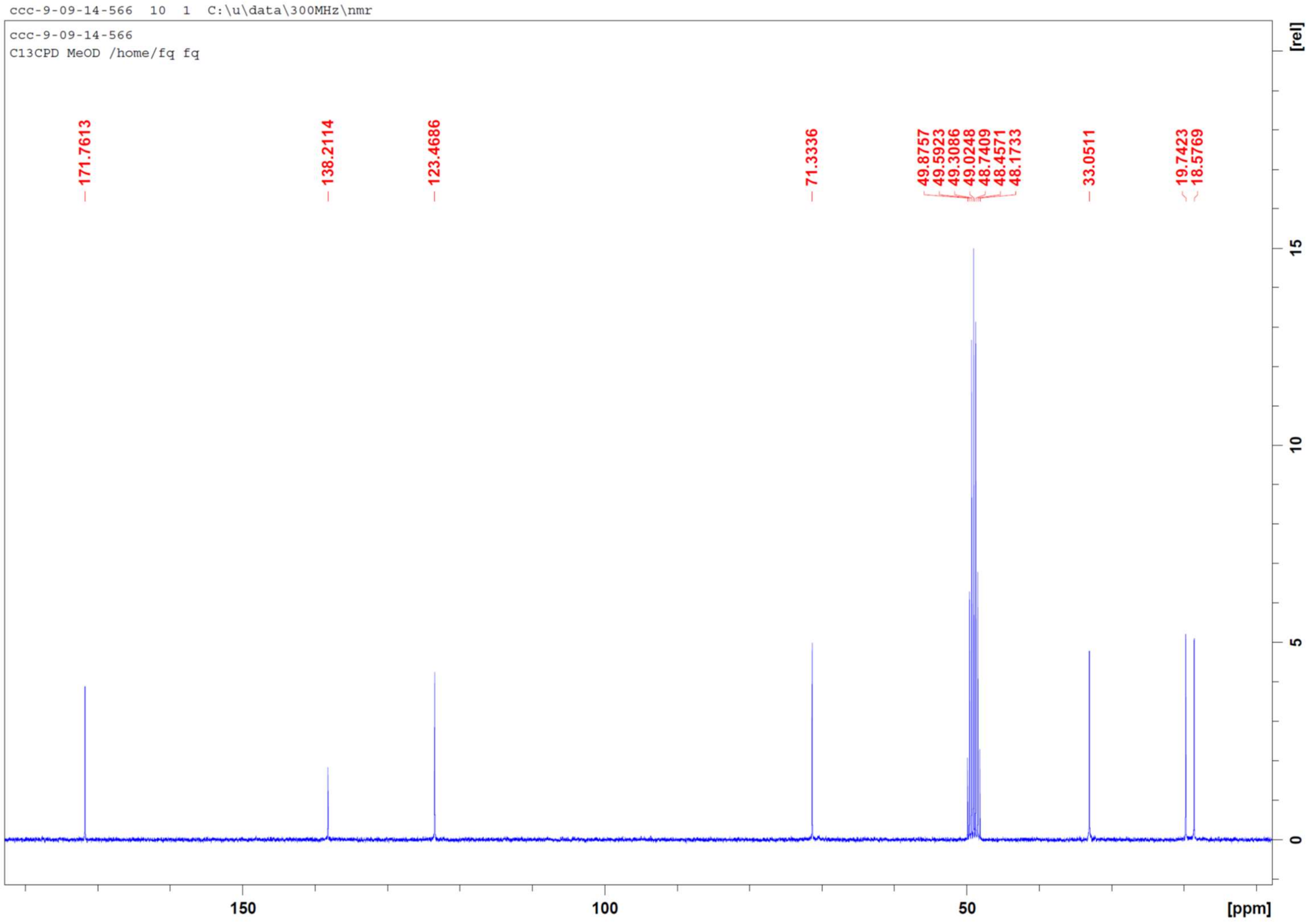

MS spectrum of **1c**.

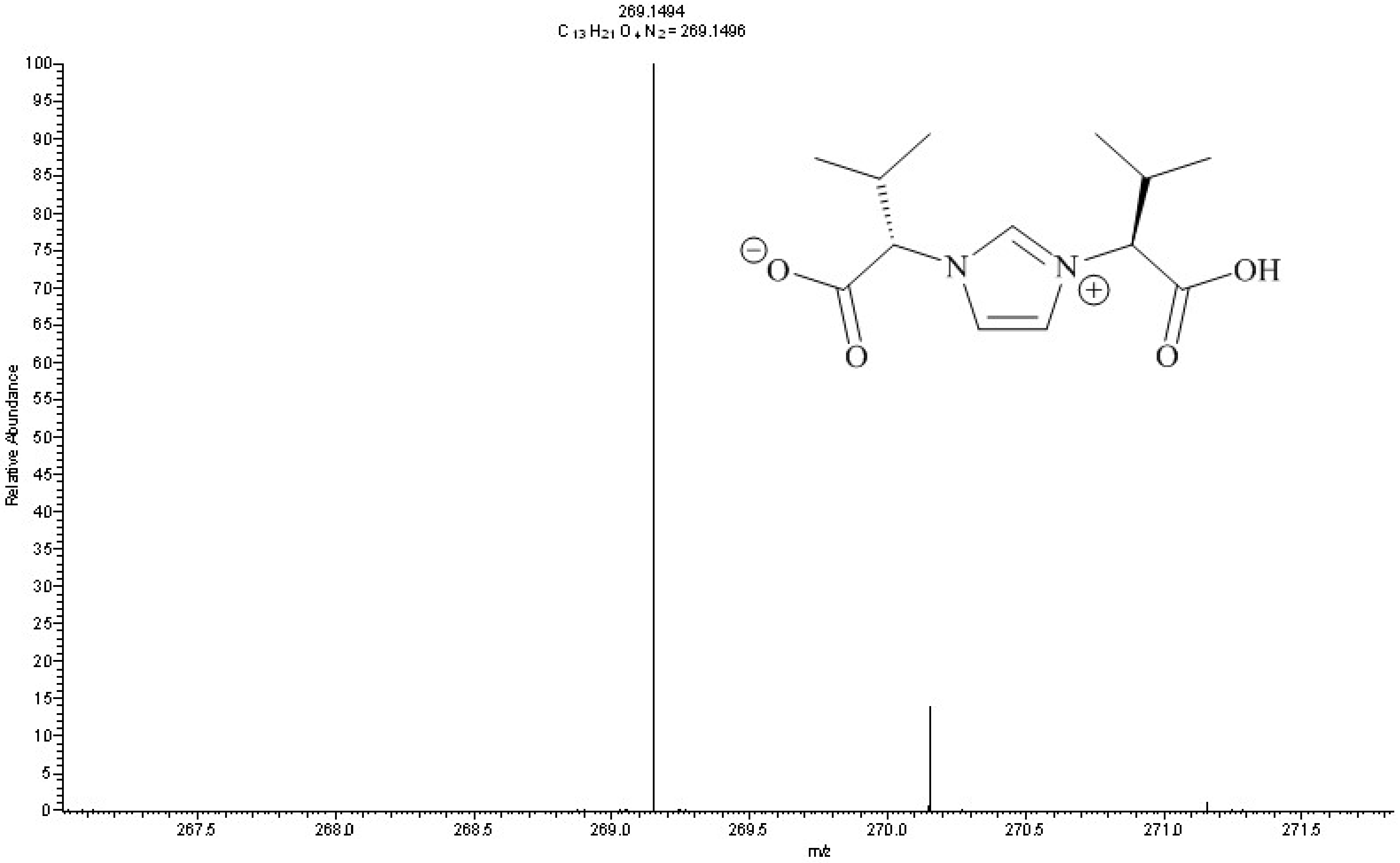

^{1}H NMR spectrum of **1d**.

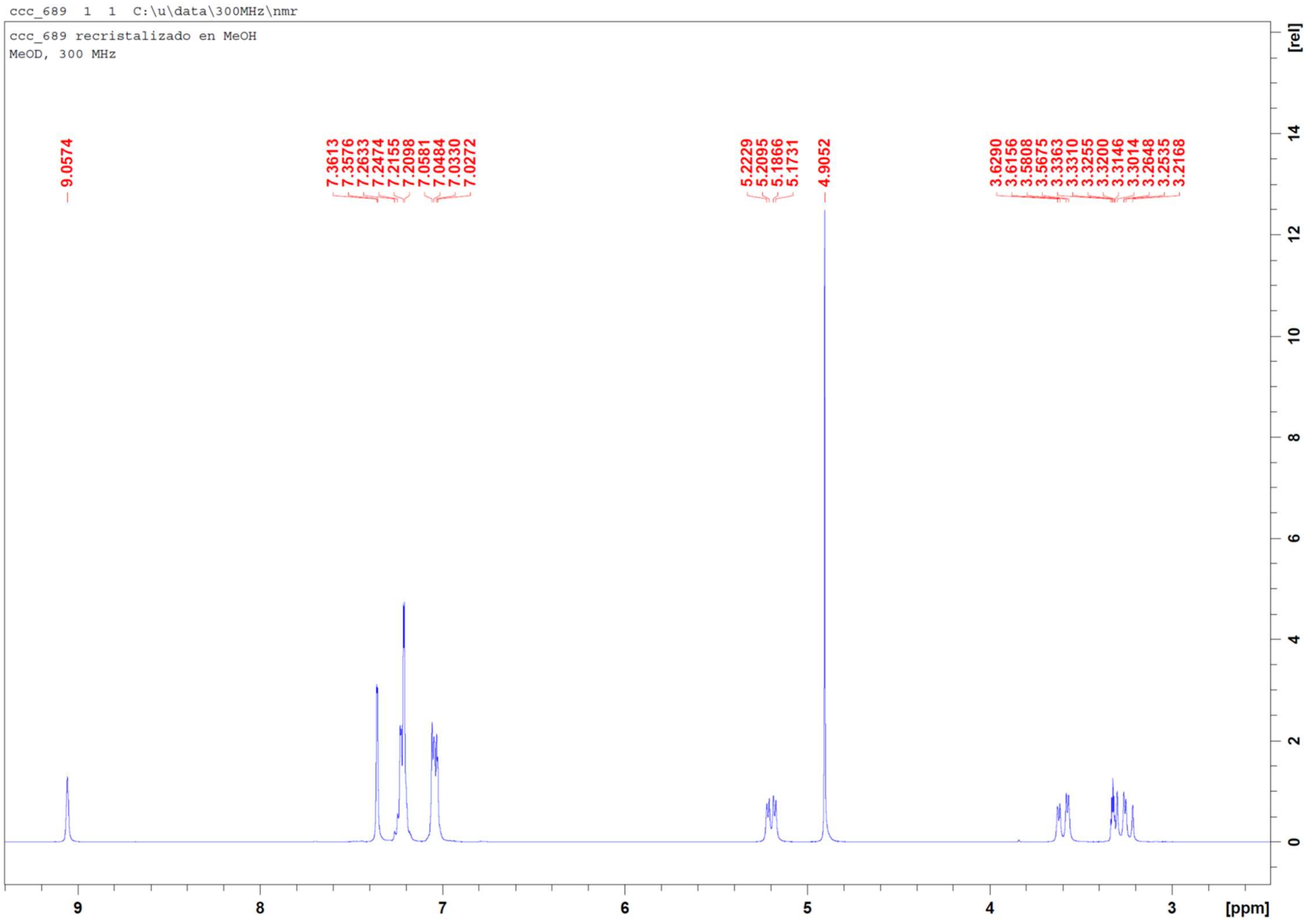

$^{13}C\{^{1}H\}$ NMR spectrum of **1d**.

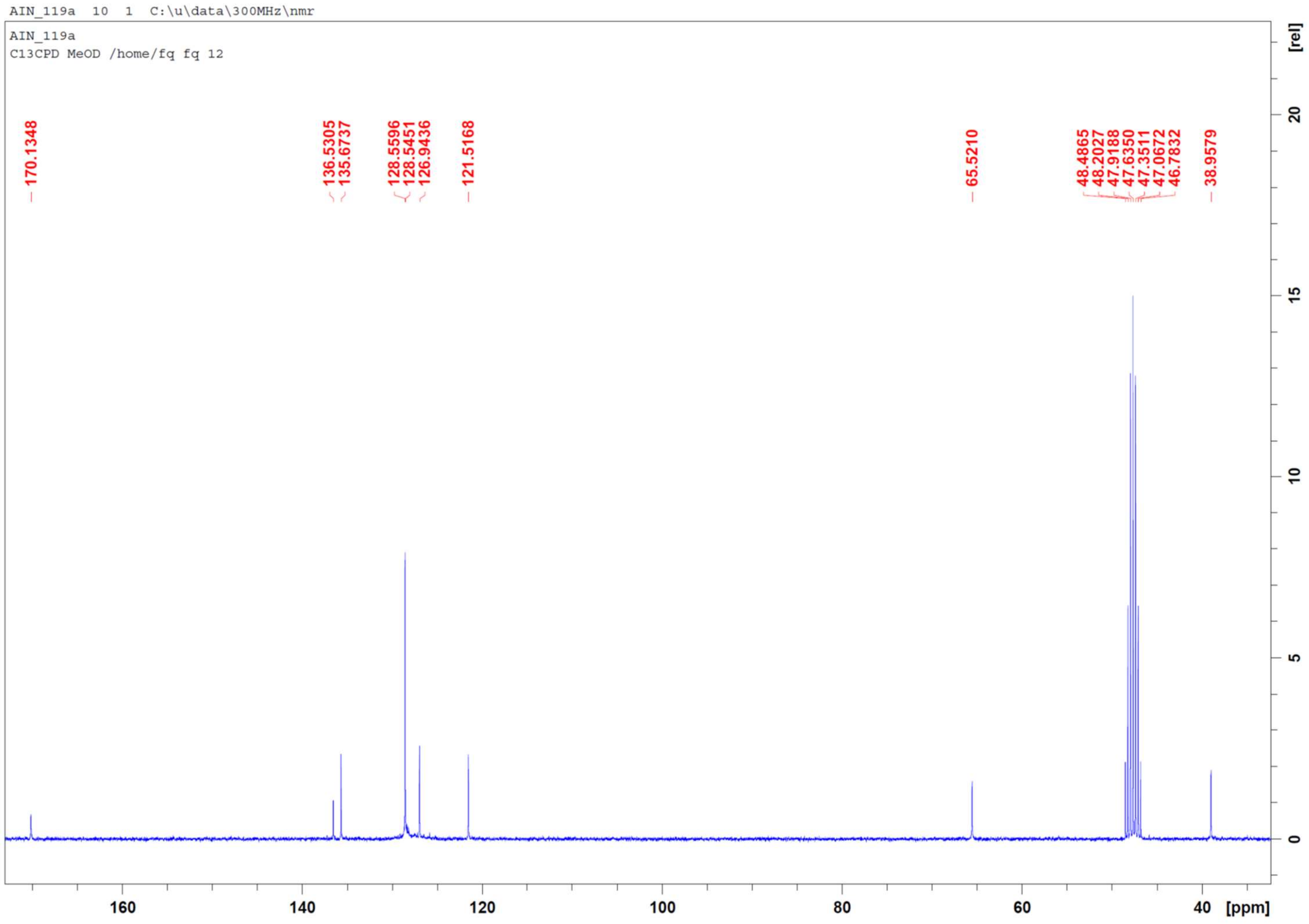

MS spectrum of **1d**.

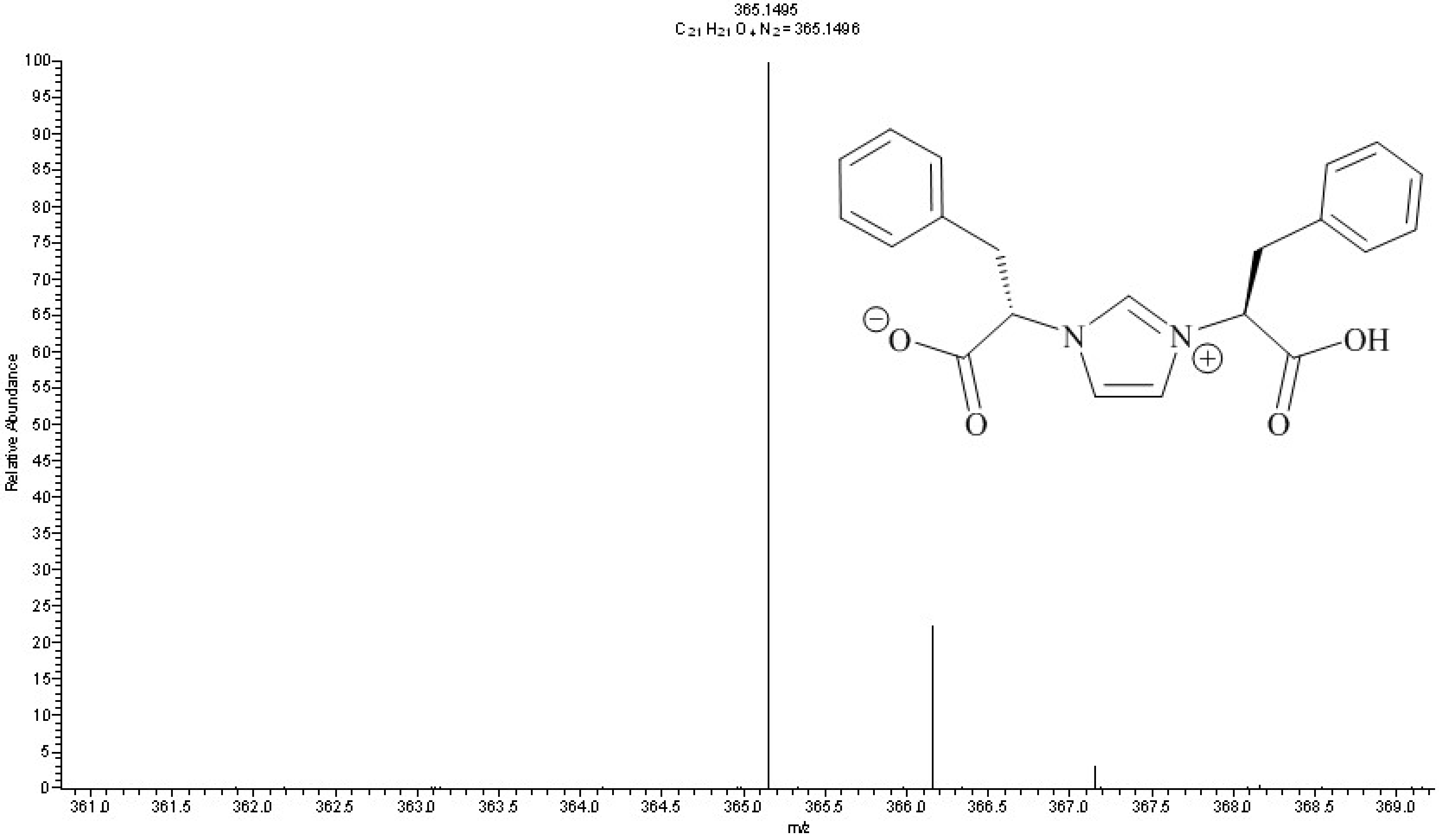

^{1}H NMR spectrum of **1e**.

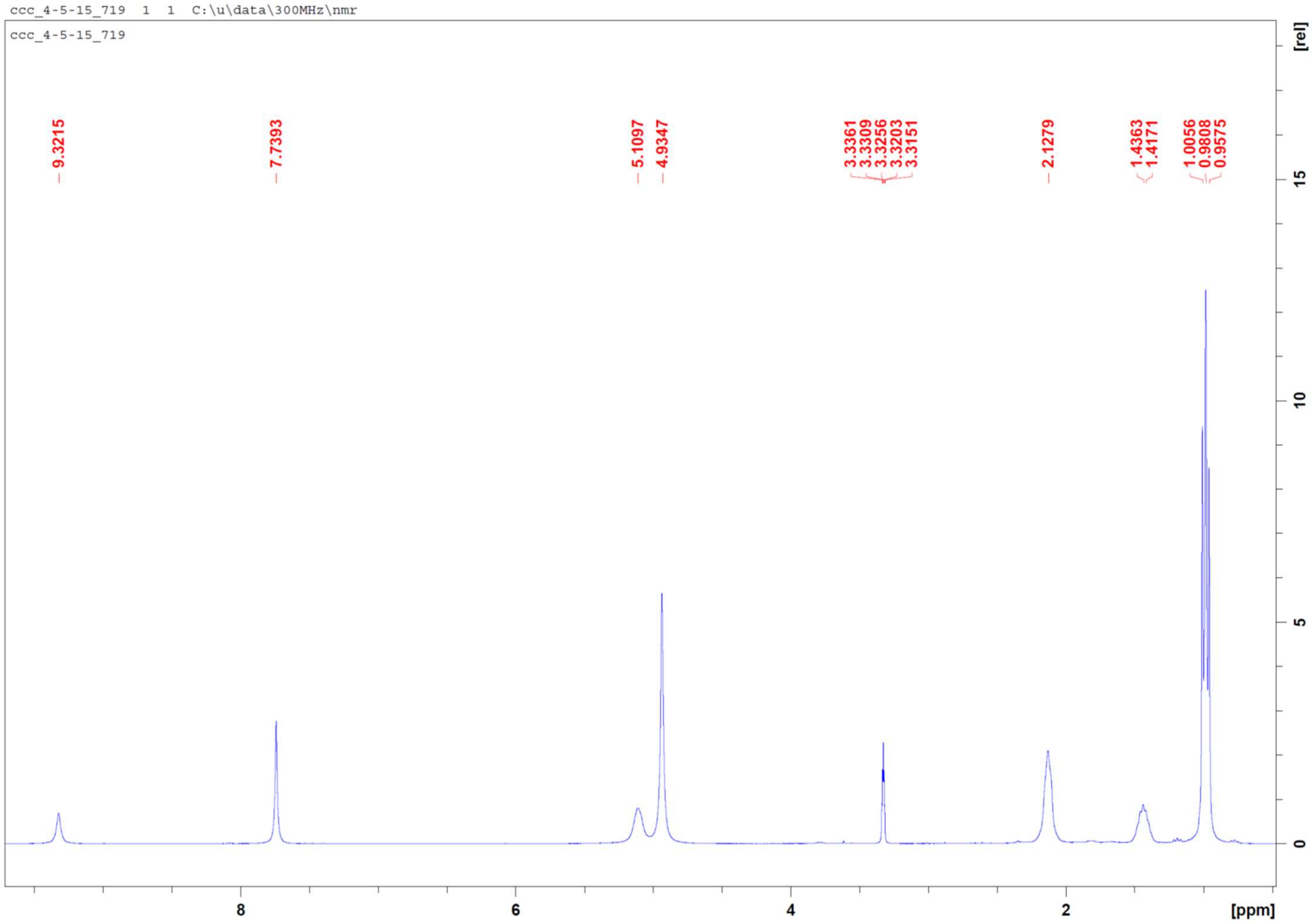

$^{13}C\{^{1}H\}$ NMR spectrum of **1e**.

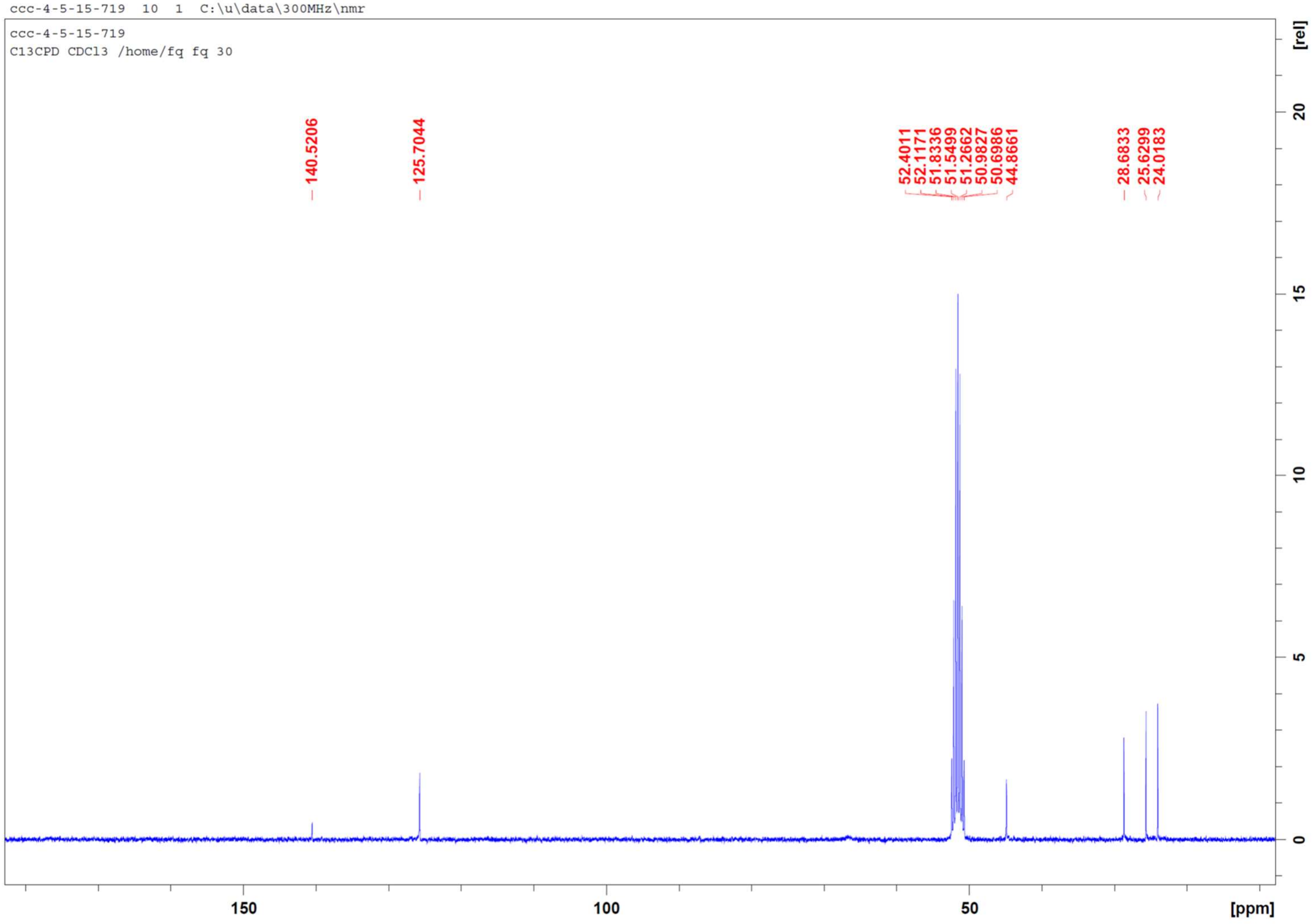

MS spectrum of **1e**.

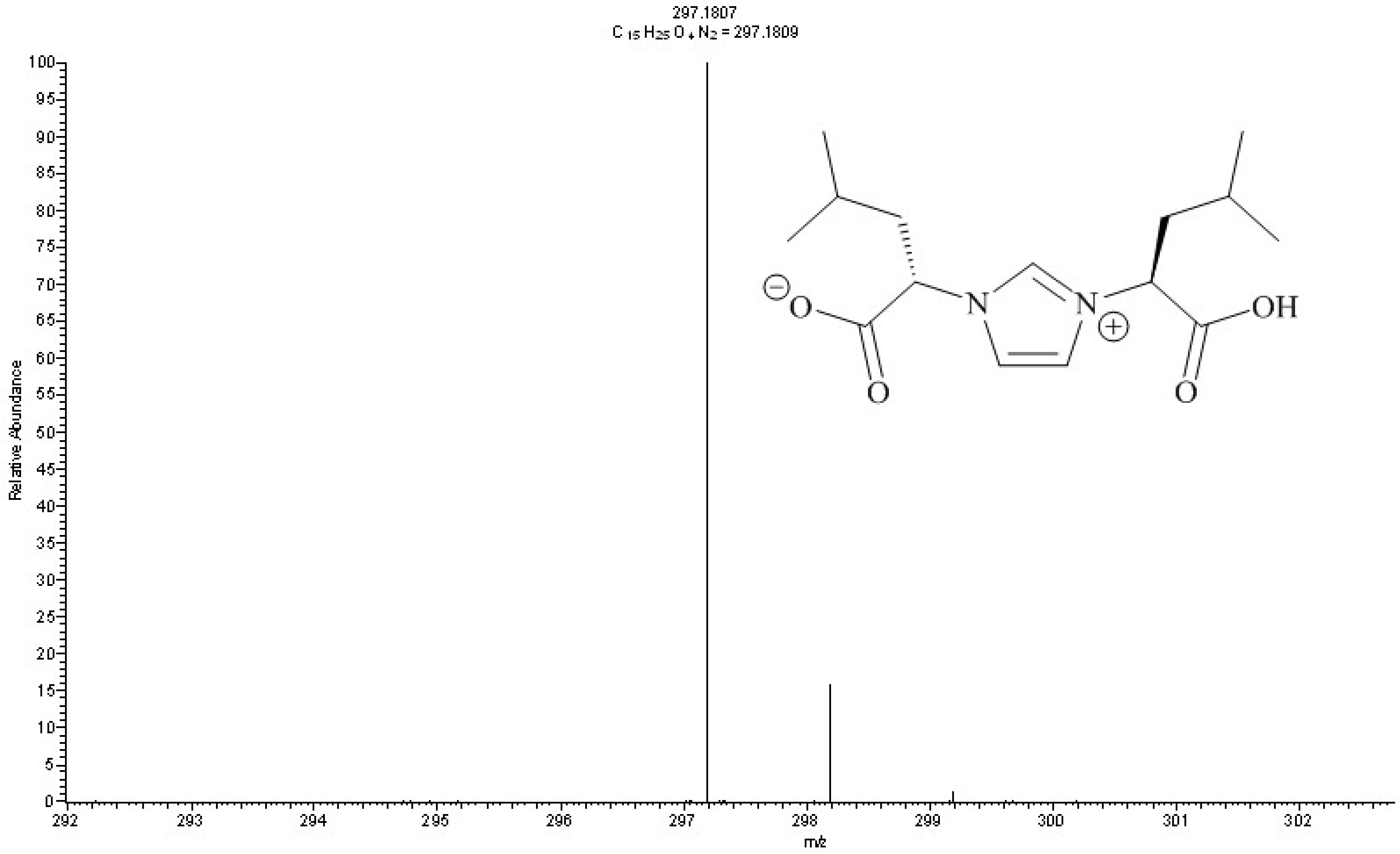

^{1}H NMR spectrum of **1f**.

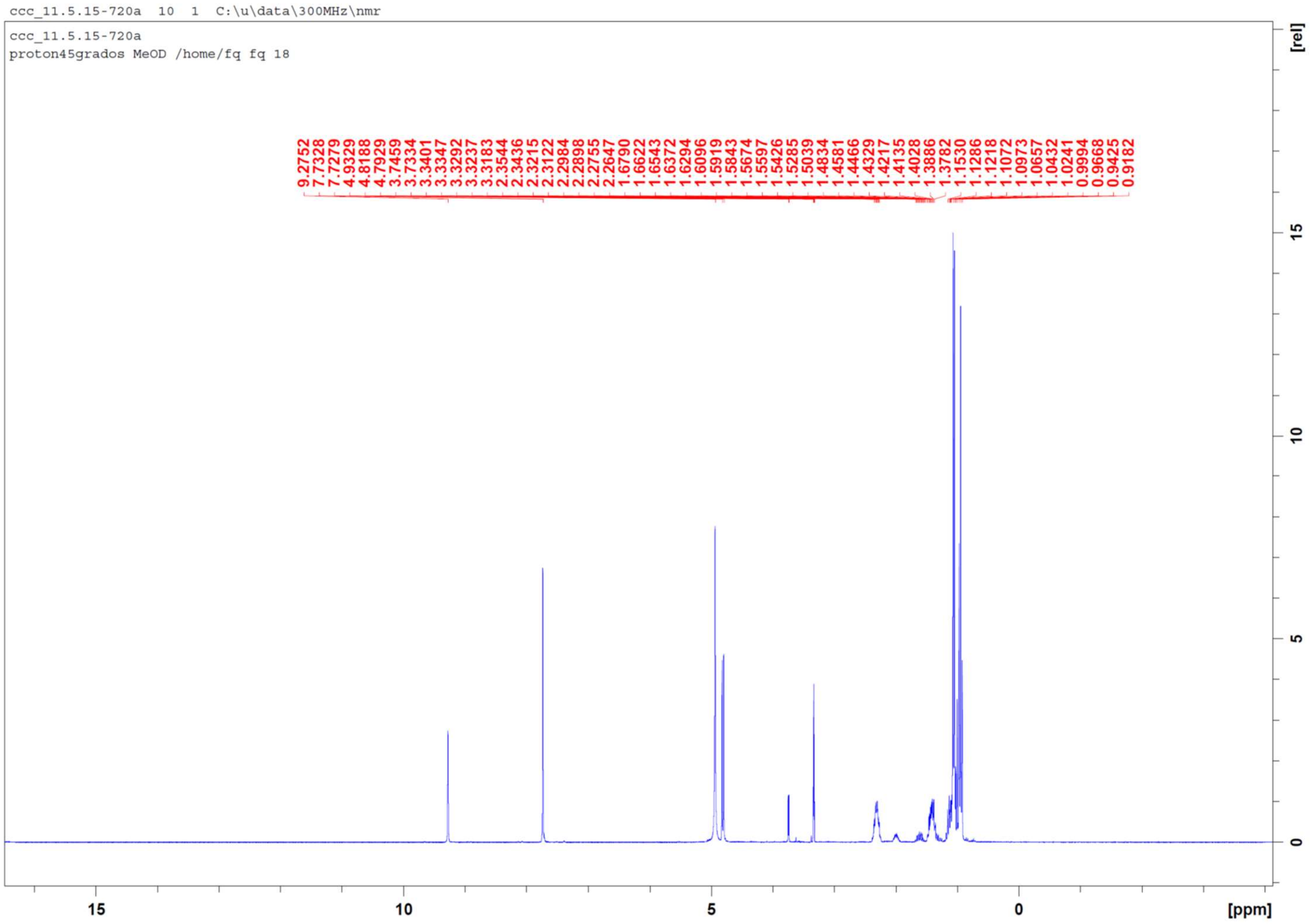

$^{13}C\{^{1}H\}$ NMR spectrum of **1f**.

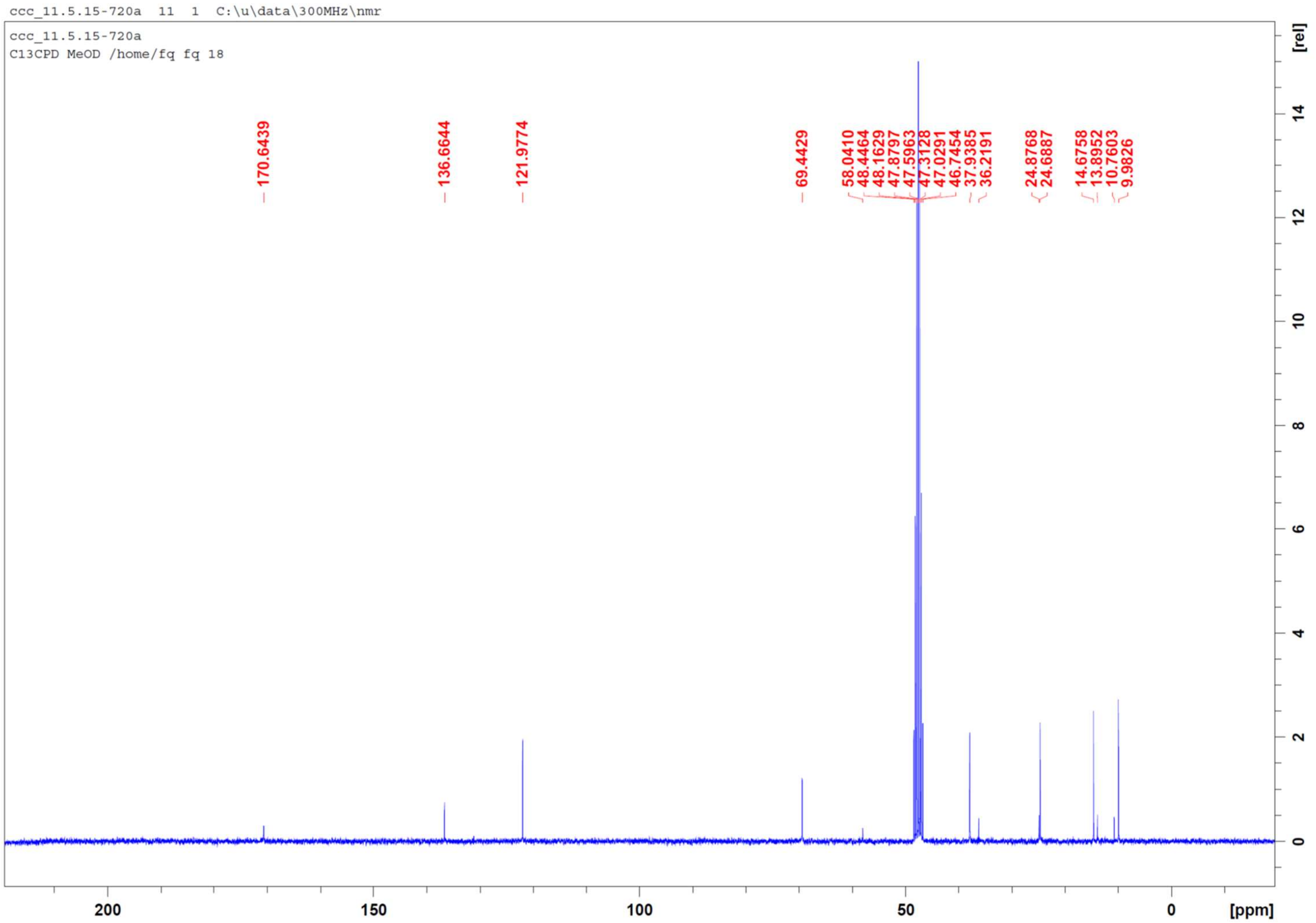

MS spectrum of **1f**.

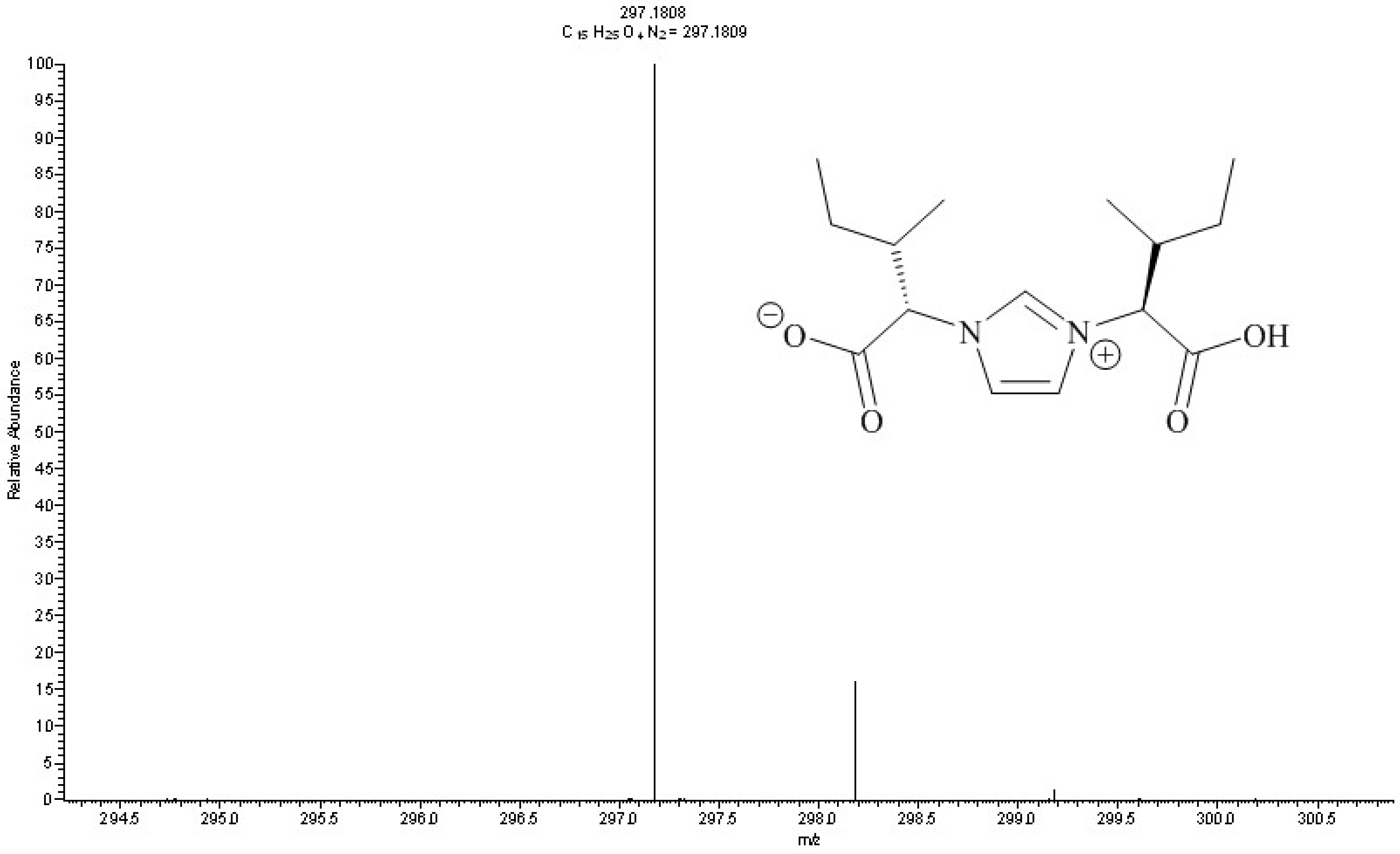

Fig. S7. Comparison of the IR spectrum of complex Na{[Mo(O)(O_2)$_2$(H_2O)]$_2$(μ-L^{iPr})} (experimental) with the calculated IR spectrum of the {[Mo(O)(O_2)$_2$(H_2O)]$_2$(μ-L^{iPr})}$^-$ anion, **2c**.

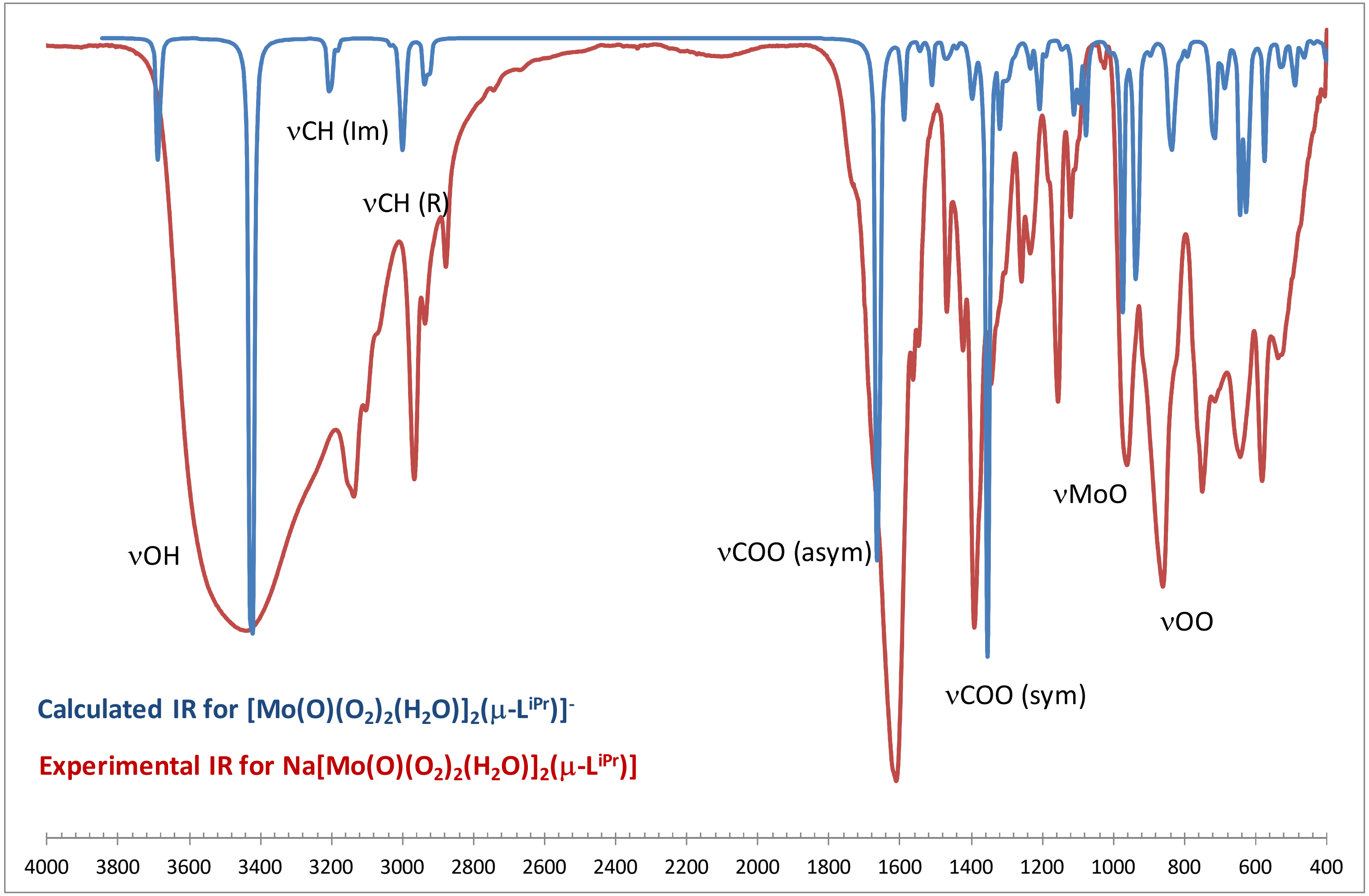

Determination of the stereoselectivity factor ($E = k_S'/k_R'$) of kinetic resolution.

Fig. S8. Schematic diagram showing the four rate constants operating in the catalytic oxidation of PhMeS.

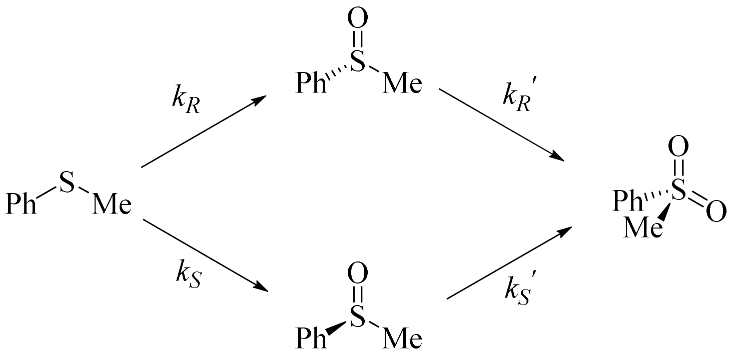

The resolution of two enantiomers in a racemate by kinetic resolution is an example of two competing reactions (Fig. S10). *E* is the stereoselectivity factor, which is the ratio between the specificity constants for the two competing enantiomers. Assuming that the reaction is irreversible, *E* can be expressed as described in the next equation (reference 73 of the manuscript):

$$E = \frac{k_S'}{k_R'} = \frac{\ln[(1-c)(1-ee)]}{\ln[(1-c)(1+ee)]}$$

where *ee* is the enantiomeric excess and *c* the conversion. Thus, provided that the data on the sulfoxide conversions and *ee*s in a series of experiments with different oxidant:substrate ratio have been collected, one can determinate from the experimental data a stereoselectivity factor ($E = k_S'/k_R'$) of 2.8 (see the following Table and Fig. S11).

**Conversion (%)**	**ee (%)**	***E*** **=** k_S'/k_R'
0.0	0.0	
20.0	10.4	2.68
45.0	29.0	2.74
68.0	56.0	2.82
89.0	89.0	2.81

Fig. S9. Sulfoxide conversion, *c*, *versus* the sulfoxide enantioselectivy, *ee*, in the kinetic resolution of racemic PhMeSO. Catalyst: $[MoO(O_2)_2(H_2O)_n]$/**1c**/$[PPh_4]Br$, CH_3Cl, 0 °C, sulfoxide:Mo ratio of 100:2.5.

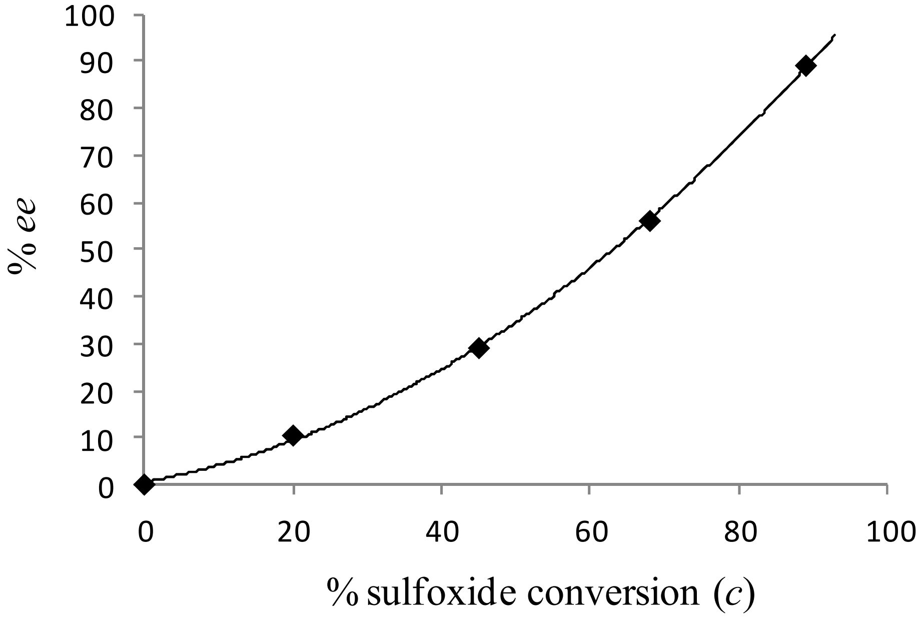

Fig. S10. Optimized structures of compounds $\{[Mo(O)(O_2)_2(H_2O)]_2(\mu\text{-}L^R)\}^-$ **2**.

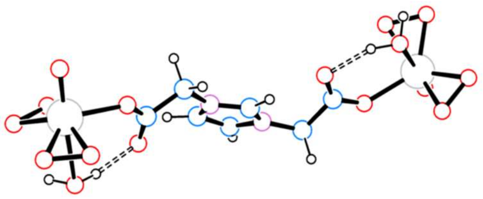

2a

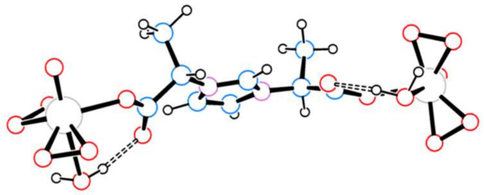

2b

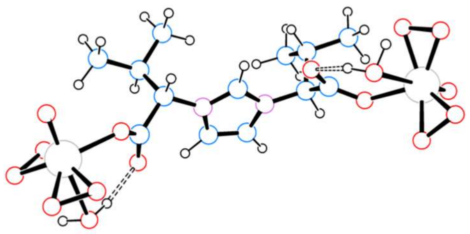

2c

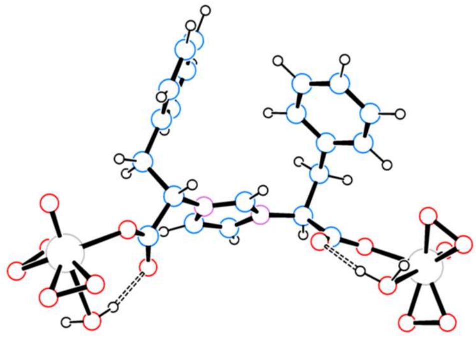

2d

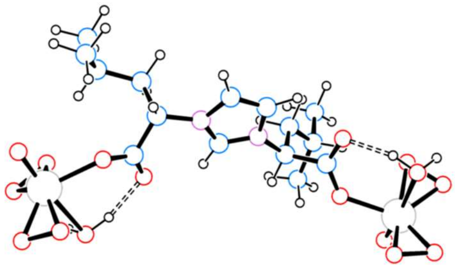

2e

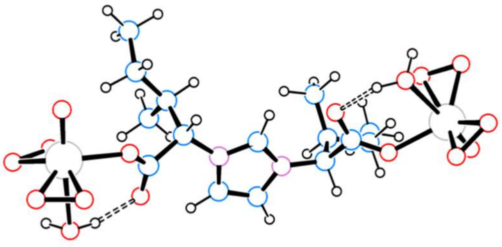

2f

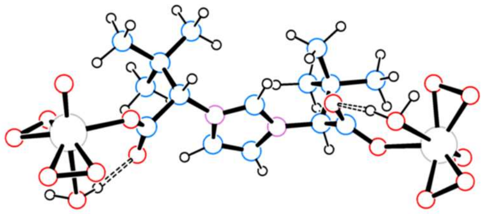

2g

Fig. S11. Optimized structures of the transition states for the oxido-transfer to PhMeS from $[Mo(O)(O_2)_2(H_2O)(\kappa^1\text{-}O\text{-}L^R)]^-$ (R = H, top; and ^{i}Pr, bottom) complexes.

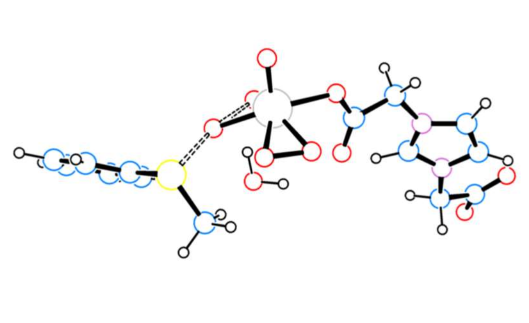	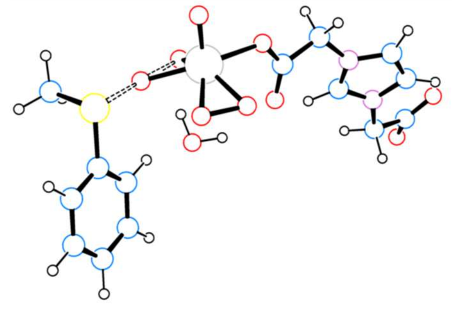	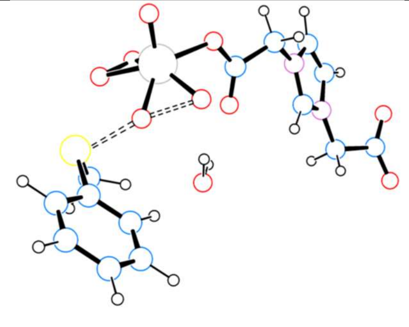	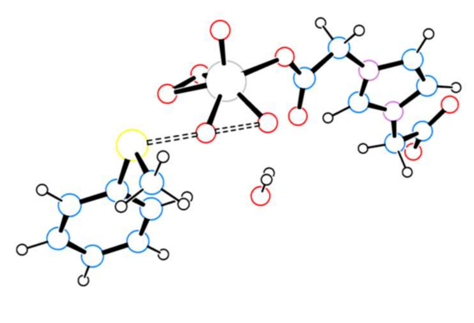
**TS_a1**	**TS_a2**	**TS_a3**	**TS_a4**

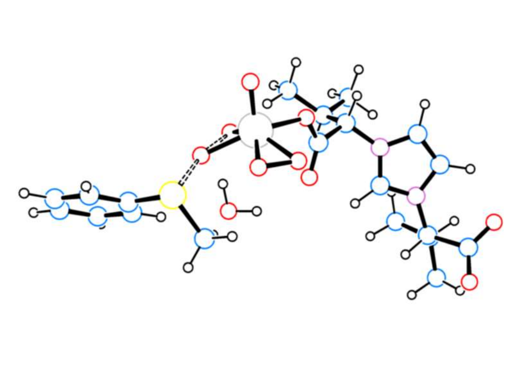	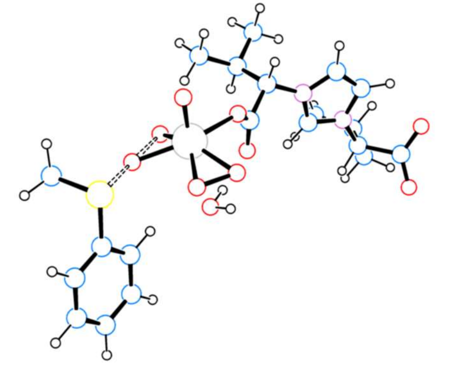	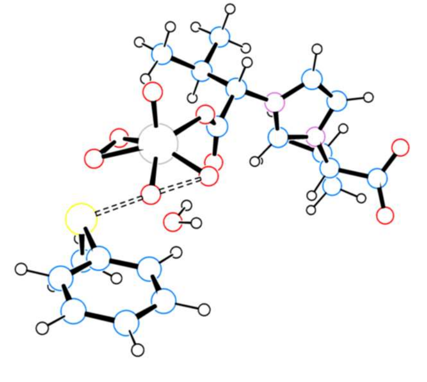	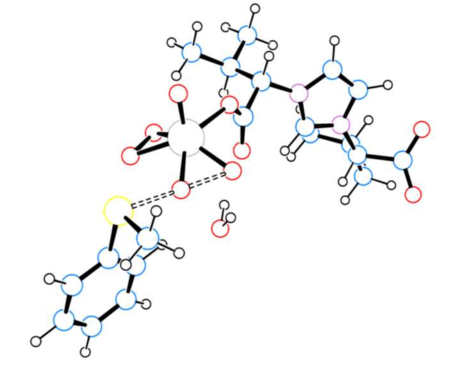
**TS_c1**	**TS_c2**	**TS_c3**	**TS_c4**

Fig. S12. Selected chiral HPLC diagrams† of optical active sulfoxides with different *ee* (entries 9-13 of Table 1) and comparison with racemic¥ mixtures.

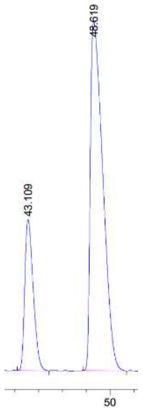	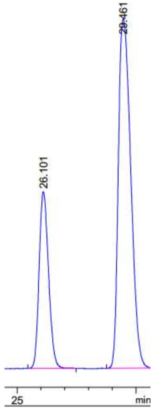	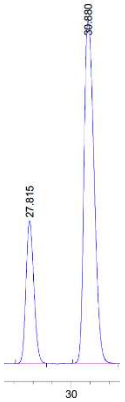
55 % *ee* of (*R*)-(*p*-Me-C_6H_4)MeSO	44 % *ee* of (*R*)-(*p*-Cl-C_6H_4)MeSO	51 % *ee* of (*R*)-(*p*-Br-C_6H_4)MeSO

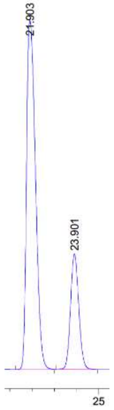	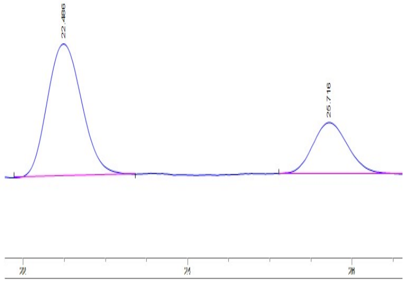
53 % *ee* of (*R*)-Ph($PhCH_2$)SO	43 % *ee* of (*S*)-Ph($HOCH_2CH_2$)SO

† For HPLC conditions see Materials and Methods section (4.1).

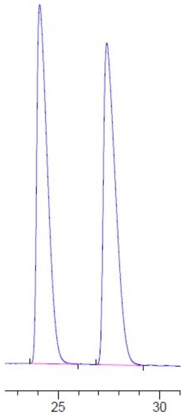	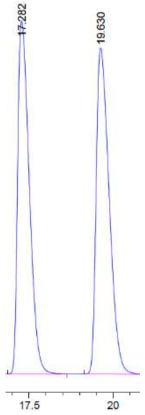	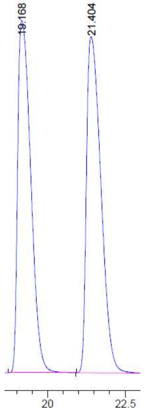
Racemic (*R*)-(*p*-Me-C_6H_4)MeSO (~1%)	Racemic (*R*)-(*p*-Cl-C_6H_4)MeSO (<2%)	Racemic (*R*)-(*p*-Br-C_6H_4)MeSO (<2%)

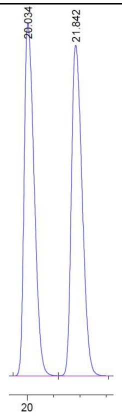	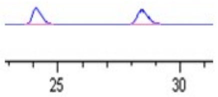
Racemic (*R*)-Ph($PhCH_2$)SO (<1%)	Racemic (*S*)-Ph($HOCH_2CH_2$)SO (~1%)

¥ Racemic mixtures were obtained using **1a** and the experimental procedure described in Materials and Methods section (4.5).

Table S1. Calculated energies (Hartree) of the transition states for the oxido-transfer to PhMeS from $[Mo(O)(O_2)_2(H_2O)(\kappa^1\text{-}O\text{-}L^R)]^-$ (R = H, ^{i}Pr) complexes.

	TS_a1	TS_a2	TS_a3	TS_a4
Eo	-1870,886790000	-1870,885383000	-1870,886791000	-1870,885398000
Et	-1870,856423000	-1870,854676000	-1870,856424000	-1870,854723000
H	-1870,855479000	-1870,853731000	-1870,855480000	-1870,853779000
G	-1870,954134000	-1870,954345000	-1870,954138000	-1870,954010000

	TS_c1	TS_c2	TS_c3	TS_c4
Eo	-2106,617163000	-2106,613519000	-2106,614014000	-2106,614621000
Et	-2106,578609000	-2106,575164000	-2106,575548000	-2106,575836000
H	-2106,577665000	-2106,574219000	-2106,574604000	-2106,574892000
G	-2106,692697000	-2106,688982000	-2106,690186000	-2106,691351000

Table S2. Coordinates of the optimized structures.

{[Mo(O)(O_2)$_2$(H_2O)]$_2$(μ-L^H)}$^-$ anion, 2a

Mo	6.34552900	-0.63764900	-0.10319200
O	6.49558800	-1.95539900	-1.16627900
O	6.13688100	-1.43780600	1.66148400
O	7.50625500	-1.07202500	1.39378900
O	6.31864000	0.92499700	-1.29963600
O	7.63816000	0.68709300	-0.75261800
O	4.19819400	-0.60243800	-0.20287600
C	3.33898800	0.33607100	-0.12144500
O	3.46260200	1.49926400	0.27598800
C	1.96802000	-0.14511600	-0.65795900
H	1.71630700	-1.10528900	-0.20664500
N	0.87227200	0.79409300	-0.38890600
C	0.88696200	2.15563900	-0.63509300
C	-0.32307500	0.45872000	0.11382600
C	-0.33365700	2.63448600	-0.26768900
H	1.77081000	2.64943800	-0.99857500
H	-0.66505800	-0.53313500	0.35605300
H	-0.73300700	3.63528000	-0.28526400
N	-1.07675700	1.56264000	0.19937600
C	-2.44895100	1.64574600	0.71559700
H	-2.42576300	1.65716500	1.80976200
C	-3.36996300	0.48445700	0.26705600
O	-4.58832800	0.78103600	0.48377700
O	-2.86767100	-0.55504500	-0.17839500
Mo	-6.47889800	-0.14031500	0.02451500
O	-7.32936100	1.33148900	0.05079500
O	-6.49417800	-0.75996000	1.87254000
O	-7.55358900	-1.35441500	1.09534600
O	-5.84627700	-0.28076100	-1.83330200
O	-7.06186400	-1.03693300	-1.61719000
O	5.98278800	1.43323600	1.36917300
H	5.11013400	1.69505700	1.00938900
H	6.63313700	1.91619900	0.83877100
O	-5.03456300	-2.24999700	-0.11927200
H	-5.40017000	-2.45071800	-0.99297800
H	-4.17119700	-1.82225500	-0.29168000
H	-2.87485600	2.59048300	0.37815300
H	2.05638600	-0.29360200	-1.73822700

{[Mo(O)(O_2)$_2$(H_2O)]$_2$(μ-L^{Me})}$^-$ anion, 2b

Mo	-6.27071700	-0.65045500	0.19539600
O	-6.56972400	-0.92336600	1.84734900
O	-5.89949300	-2.41432400	-0.54456500
O	-7.27816800	-2.03105800	-0.72833700
O	-6.33402800	1.31286700	0.03692700
O	-7.59013300	0.69836600	-0.33789900
O	-4.15129800	-0.43709800	0.46292100
C	-3.28114500	0.32166700	-0.08440900
O	-3.32438200	0.90579700	-1.17301000
C	-2.04781300	0.48333600	0.84527400
H	-1.67906200	-0.52038700	1.07132900
C	-2.44080700	1.19481000	2.14806100
H	-2.81223100	2.20372600	1.94441400
N	-0.92819700	1.19120300	0.18184100
C	-1.00674100	2.32719800	-0.60519400
C	0.36298300	0.87166100	0.34878200
C	0.27191700	2.67991400	-0.91313700
H	-1.95622200	2.72747700	-0.91304400
H	0.76408300	0.02757100	0.88542000
H	0.65216100	3.48917300	-1.51508900

N	1.11731100	1.75934300	-0.31410100
C	2.59619900	1.86907700	-0.34642500
H	2.84377000	2.24009100	-1.34359600
C	3.08103800	2.87017900	0.71426000
H	2.85614900	2.50278900	1.71987800
C	3.33310800	0.51878500	-0.16665000
O	4.54325800	0.62462300	-0.55610500
O	2.75438000	-0.45438400	0.33557300
Mo	6.34993400	-0.44335000	-0.09054400
O	7.32952200	0.94468700	-0.17626800
O	6.29768900	-1.12944000	-1.91364100
O	7.30205400	-1.79020400	-1.11655000
O	5.72597200	-0.44866900	1.77711400
O	6.86360400	-1.32237500	1.58396000
O	-5.67228500	-0.07512400	-2.22214200
H	-4.83788400	0.40508600	-2.03809100
H	-6.35893300	0.60725200	-2.23735300
O	4.71877100	-2.39021500	0.13848200
H	5.08770500	-2.62161700	1.00323000
H	3.90893800	-1.87576600	0.33335500
H	-3.23915500	0.62660400	2.62757200
H	-1.58672000	1.26417700	2.82857300
H	4.16253400	2.98267000	0.62018500
H	2.60563200	3.84673500	0.58139200

{[Mo(O)(O_2)$_2$(H_2O)]$_2$(μ-L^{iPr})}$^-$ anion, 2c

Mo	5.86982600	-1.19200400	-0.26790300
O	6.05227900	-1.23430900	-1.95797500
O	5.08620400	-2.91259400	0.19585300
O	6.52124300	-2.91442000	0.34934800
O	6.42360500	0.63986200	0.20962600
O	7.50119800	-0.31114900	0.37814900
O	3.87596400	-0.39715300	-0.35203000
C	3.27966700	0.47998700	0.35935900
O	3.49837200	0.81249800	1.53338900
C	2.19126500	1.24449300	-0.43456700
H	1.89637600	0.63653800	-1.29262300
C	2.73795500	2.61832600	-0.92078700
H	2.99022000	3.18893600	-0.01695200
C	4.01980800	2.43765300	-1.75147700
H	4.84357300	2.01371300	-1.17355300
H	3.84482000	1.78233500	-2.61172100
H	4.34439300	3.41146000	-2.13223100
C	1.68357600	3.39814200	-1.72014200
H	0.78154300	3.61287500	-1.13833600
H	2.09737200	4.35794500	-2.04414100
H	1.38600500	2.84808100	-2.62117100
N	0.96680500	1.42877900	0.38306400
C	0.90013400	1.89439500	1.68616700
C	-0.28182900	1.26788900	-0.07875300
C	-0.42232900	2.00639900	1.99599200
H	1.79904800	2.03935100	2.26031200
H	-0.57366700	0.87946200	-1.04023500
H	-0.90798200	2.31489000	2.90716500
N	-1.14785200	1.61494800	0.88416500
C	-2.62377200	1.67180800	0.76478500
H	-3.00331400	1.76052500	1.78476700
C	-3.06765400	2.90560400	-0.07266600
H	-2.62866700	2.77475100	-1.07115800
C	-2.54301900	4.21732800	0.53046600
H	-2.96056100	4.38232700	1.53131900
H	-1.45172400	4.24262700	0.61011200
H	-2.84672300	5.06277600	-0.09440300
C	-4.59726600	2.95182900	-0.22157800
H	-4.88109900	3.86107300	-0.76128300
H	-4.99630100	2.09785400	-0.77281300

H	-5.08986400	2.97468500	0.75666700
C	-3.17633200	0.36489000	0.14702800
O	-4.23079000	-0.04434500	0.73801300
O	-2.59696200	-0.10726300	-0.84256300
Mo	-5.92849300	-1.17824500	0.06923900
O	-7.03869700	-0.27553700	0.98942100
O	-5.34763800	-2.60785300	1.25730200
O	-6.41390400	-3.03258600	0.38254500
O	-5.77808700	-0.14914100	-1.60694400
O	-6.71764900	-1.24326200	-1.72506700
O	5.50204600	-0.92769800	2.24703300
H	4.81934000	-0.22541000	2.19794700
H	6.34311300	-0.45581500	2.33203000
O	-4.15338300	-2.32702400	-1.33888100
H	-4.66354900	-2.20101700	-2.15183800
H	-3.48263400	-1.61226300	-1.34529800

{[Mo(O)(O_2)$_2$(H_2O)]$_2$(μ-L^{iBu})}$^-$ anion, 2e

Mo	-6.16279100	-1.09275000	-0.00193800
O	-6.19520200	-1.52382800	1.64278200
O	-5.68191000	-2.72992100	-0.93944000
O	-7.10999800	-2.52388500	-0.91213100
O	-6.48194700	0.85323900	0.02457000
O	-7.69803800	0.10888700	-0.22417000
O	-4.07326900	-0.58410100	0.03625400
C	-3.39854200	0.32615400	-0.55432700
O	-3.63876400	0.89787400	-1.62808200
C	-2.17929500	0.77996000	0.28204000
H	-1.90269400	-0.03171000	0.95509100
C	-2.52180200	2.06036900	1.07839900
H	-2.75444500	2.84890200	0.35239600
C	-3.68381900	1.93242500	2.08597200
H	-4.59607200	1.66863200	1.53799200
N	-0.98235800	1.03309800	-0.56192000
C	-0.93280500	1.64437500	-1.80389100
C	0.27121300	0.78253800	-0.15594300
C	0.38422000	1.74219900	-2.13955600
H	-1.84172300	1.89641200	-2.32299700
H	0.58205900	0.30377500	0.75860600
H	0.85655200	2.13605100	-3.02481300
N	1.12389400	1.19848000	-1.10315100
C	2.60781000	1.22008800	-1.04987100
H	2.94002300	1.13112600	-2.08456600
C	3.07177000	2.56528300	-0.44046700
H	2.69011200	2.60816000	0.58707300
C	4.59391300	2.81788900	-0.43381100
H	5.07693000	2.04008100	0.16866000
C	3.19332100	0.03358200	-0.25354800
O	4.28905900	-0.38179900	-0.76038900
O	2.61856800	-0.34811500	0.77759200
Mo	6.05390900	-1.27516500	0.08781300
O	7.09729300	-0.47431000	-0.99145800
O	5.57712100	-2.93041700	-0.82024200
O	6.66951900	-3.11656300	0.10406500
O	5.82939500	0.02510900	1.55417500
O	6.84364400	-0.96243700	1.85437800
O	-5.98399300	-0.28115300	-2.41360900
H	-5.16881400	0.25534300	-2.31377200
H	-6.71364700	0.34084900	-2.28049200
O	4.35822500	-2.27534800	1.69162700
H	4.82467500	-1.93345600	2.46775900
H	3.61789500	-1.65020100	1.54287200
H	2.57720900	3.37234300	-0.99979800
H	-1.61356900	2.37246200	1.61376300
C	-3.43318200	0.84458400	3.14101000
H	-4.24307600	0.83861500	3.87685900

H	-3.40318500	-0.15379900	2.69694500
H	-2.49287500	1.02256200	3.68026300
C	-3.91979300	3.29818100	2.74972700
H	-4.13099800	4.07585300	2.00725300
H	-4.77335600	3.25023100	3.43314200
H	-3.04409300	3.61550600	3.33075600
C	4.87098000	4.16913300	0.24372700
H	5.94823000	4.34915700	0.31318300
H	4.42915000	4.99848000	-0.32400900
H	4.46212600	4.20125300	1.25961200
C	5.20541100	2.77445600	-1.84196000
H	5.14302600	1.77570300	-2.28175200
H	4.71133000	3.49143200	-2.51226600
H	6.26754000	3.03448400	-1.80134500

{[Mo(O)(O_2)$_2$(H_2O)]$_2$(μ-L$^{sec\text{-}Bu}$)}$^-$ anion, 2f

Mo	-6.23347700	-1.06203700	0.07266600
O	-6.22031700	-1.59506900	1.68721500
O	-5.92916600	-2.66728800	-0.98640500
O	-7.33393100	-2.35778200	-0.86752900
O	-6.39822600	0.89701000	0.23851400
O	-7.67909000	0.26413700	0.00695600
O	-4.11596700	-0.70396800	0.02121300
C	-3.40691600	0.19515700	-0.54525600
O	-3.64822900	0.82596300	-1.58627400
C	-2.13859700	0.52228100	0.27597800
H	-1.85755400	-0.38317300	0.81665400
C	-2.39256000	1.66074500	1.31433300
C	-3.39791500	1.20126200	2.39447500
H	-4.41031000	1.19274000	1.97731600
H	-3.18010000	0.16401200	2.67448100
N	-0.97008600	0.85271500	-0.58407100
C	-0.94746100	1.51132200	-1.80317900
C	0.29449200	0.60869000	-0.20670600
C	0.36292500	1.64996000	-2.14953800
H	-1.86804900	1.76705400	-2.29886800
H	0.62576900	0.08903900	0.67740600
H	0.81489500	2.08947300	-3.02381400
N	1.12777800	1.08050400	-1.14487000
C	2.61158500	1.11768600	-1.12855700
H	2.91484100	1.03812400	-2.17434900
C	3.11380600	2.48811100	-0.56915100
C	4.61153100	2.69619200	-0.88675200
H	5.21856900	2.05769400	-0.23751500
H	4.81300100	2.36322100	-1.91200800
C	3.23310100	-0.07687300	-0.37264900
O	4.35987000	-0.41396900	-0.86954800
O	2.65295500	-0.54659700	0.61917700
Mo	6.16990600	-1.17047700	0.01257500
O	7.17928600	-0.31332800	-1.05503800
O	5.84226300	-2.87143300	-0.87645200
O	6.92404700	-2.95863800	0.07504000
O	5.81267500	0.13861700	1.44552800
O	6.88833600	-0.76503100	1.79242800
O	-6.11953300	-0.11841500	-2.29218100
H	-5.25507500	0.33898200	-2.21587700
H	-6.78199500	0.55501800	-2.08103900
O	4.53578200	-2.28592400	1.60000700
H	4.93993900	-1.87722500	2.37870300
H	3.73776100	-1.74849100	1.41181900
C	5.06324100	4.15423200	-0.73865100
H	6.11750200	4.25295400	-1.01446500
H	4.48430100	4.82498700	-1.38581300
H	4.96237900	4.51349200	0.29037900
C	-3.36905500	2.07170000	3.65659300
H	-4.06974600	1.68072000	4.40049600

H	-2.37177100	2.08647600	4.11310800
H	-3.65823300	3.10750600	3.45145900
C	2.80873800	2.66387900	0.92578800
H	3.37728400	1.95171100	1.53037000
H	3.07584900	3.67337500	1.24911200
H	1.74519900	2.52877100	1.14914100
C	-2.81128500	2.98499400	0.65844300
H	-3.77334400	2.88338700	0.14894900
H	-2.90737700	3.76646700	1.41693700
H	-2.07408000	3.33359600	-0.07197500
H	-1.42021500	1.81271500	1.80870600
H	2.54688800	3.24810900	-1.12906900

{[Mo(O)(O_2)$_2$(H_2O)]$_2$(μ-L^{tBu})}$^-$ anion, 2g

Mo	5.86982600	-1.19200400	-0.26790300
O	6.05227900	-1.23430900	-1.95797500
O	5.08620400	-2.91259400	0.19585300
O	6.52124300	-2.91442000	0.34934800
O	6.42360500	0.63986200	0.20962600
O	7.50119800	-0.31114900	0.37814900
O	3.87596400	-0.39715300	-0.35203000
C	3.27966700	0.47998700	0.35935900
O	3.49837200	0.81249800	1.53338900
C	2.19126500	1.24449300	-0.43456700
H	1.89637600	0.63653800	-1.29262300
C	2.73795500	2.61832600	-0.92078700
C	4.01980800	2.43765300	-1.75147700
H	4.84357300	2.01371300	-1.17355300
H	3.84482000	1.78233500	-2.61172100
H	4.34439300	3.41146000	-2.13223100
C	1.68357600	3.39814200	-1.72014200
H	0.78154300	3.61287500	-1.13833600
H	2.09737200	4.35794500	-2.04414100
H	1.38600500	2.84808100	-2.62117100
N	0.96680500	1.42877900	0.38306400
C	0.90013400	1.89439500	1.68616700
C	-0.28182900	1.26788900	-0.07875300
C	-0.42232900	2.00639900	1.99599200
H	1.79904800	2.03935100	2.26031200
H	-0.57366700	0.87946200	-1.04023500
H	-0.90798200	2.31489000	2.90716500
N	-1.14785200	1.61494800	0.88416500
C	-2.62377200	1.67180800	0.76478500
H	-3.00331400	1.76052500	1.78476700
C	-3.06765400	2.90560400	-0.07266600
C	-2.54301900	4.21732800	0.53046600
H	-2.96056100	4.38232700	1.53131900
H	-1.45172400	4.24262700	0.61011200
H	-2.84672300	5.06277600	-0.09440300
C	-4.59726600	2.95182900	-0.22157800
H	-4.88109900	3.86107300	-0.76128300
H	-4.99630100	2.09785400	-0.77281300
H	-5.08986400	2.97468500	0.75666700
C	-3.17633200	0.36489000	0.14702800
O	-4.23079000	-0.04434500	0.73801300
O	-2.59696200	-0.10726300	-0.84256300
Mo	-5.92849300	-1.17824500	0.06923900
O	-7.03869700	-0.27553700	0.98942100
O	-5.34763800	-2.60785300	1.25730200
O	-6.41390400	-3.03258600	0.38254500
O	-5.77808700	-0.14914100	-1.60694400
O	-6.71764900	-1.24326200	-1.72506700
O	5.50204600	-0.92769800	2.24703300
H	4.81934000	-0.22541000	2.19794700
H	6.34311300	-0.45581500	2.33203000
O	-4.15338300	-2.32702400	-1.33888100

H	-4.66354900	-2.20101700	-2.15183800
H	-3.48263400	-1.61226300	-1.34529800
C	-2.45226260	2.72216847	-1.47239627
H	-3.20955271	2.85946732	-2.21574055
H	-1.67507478	3.44313704	-1.61759762
H	-2.04458332	1.73644912	-1.55639009
C	3.09168904	3.41845358	0.34659932
H	3.78945243	2.86180588	0.93665830
H	3.52792909	4.35467680	0.06717008
H	2.20346172	3.59481061	0.91655582

Transition States

TS_a1

Mo	1.34302700	-1.82059900	-0.24724000
O	1.89352800	-3.18352100	-1.11111200
O	0.63548100	-2.33730300	1.50627100
O	1.18151200	-0.39067500	-1.41312000
O	2.04481300	-2.05120100	1.59018200
O	2.68929400	-0.34126500	-0.36708500
S	4.17410600	-0.02770200	1.10694000
C	3.01549800	0.77243000	2.26362600
H	2.22164300	1.27098300	1.69829000
H	2.58498500	-0.04957600	2.83536600
C	4.88883100	1.36560400	0.25059300
C	4.10469700	2.43312500	-0.21005200
C	6.26741300	1.34377500	0.00318900
C	4.71743300	3.48008700	-0.89854400
H	3.03194500	2.43972800	-0.04678500
C	6.86550200	2.39113200	-0.69827200
H	6.86534700	0.51133600	0.36265500
C	6.09273300	3.46408800	-1.14559600
H	4.11018900	4.30768000	-1.25441500
H	7.93522300	2.36966700	-0.88825000
H	6.55873300	4.28265000	-1.68741000
O	-7.70142300	3.55608100	0.16724700
C	-7.47781600	2.33654600	0.16763100
O	-8.22137700	1.34132400	0.00107300
C	-5.95300000	2.03506800	0.52600000
H	-5.83816600	2.15633500	1.60757500
N	-5.45746500	0.68561300	0.16885400
C	-6.20016400	-0.48057400	0.21896800
C	-4.22222300	0.39369600	-0.25146600
C	-5.37623100	-1.48862000	-0.18702200
H	-7.25117000	-0.40394100	0.45752900
H	-3.39734200	1.07858000	-0.34434700
H	-5.55681900	-2.54436900	-0.30737900
N	-4.14256900	-0.92824400	-0.47957900
C	-2.98615000	-1.65694000	-1.00344400
H	-3.14900300	-2.71914400	-0.81869000
C	-1.62851800	-1.23812800	-0.39088500
O	-0.72672200	-2.09703800	-0.70234600
O	-1.52748400	-0.19221300	0.25059400
H	3.56005300	1.46416300	2.91023900
H	-2.92078500	-1.51384600	-2.08715900
H	-5.31045400	2.76132700	0.02756900
O	0.62141000	1.89780000	0.14686800
H	-0.13701600	1.40399600	0.49544400
H	0.93973300	1.26757700	-0.52906600

TS_a2

Mo	-1.41613000	-1.60466600	-0.09382300
O	-1.85142500	-3.25557000	-0.11307000
O	-0.96419300	-0.95948400	-1.88287100
O	-1.08919100	-0.99763200	1.62416200
O	-2.34010400	-0.69177900	-1.55551800
O	-2.81264300	-0.74375400	1.06807500
S	-4.80676000	-0.43852300	0.38973200
O	8.11225500	2.93362700	1.03197900
C	7.75941900	1.90677700	0.43322000
O	8.40318900	0.93667400	-0.03129700
C	6.18658700	1.91105500	0.16715500
H	5.99636800	2.58056000	-0.67743600
N	5.58110200	0.59805000	-0.15333600

C	6.18684000	-0.40475100	-0.88904000
C	4.36445400	0.18676600	0.21933800
C	5.29957600	-1.43858300	-0.94856100
H	7.21410500	-0.26345600	-1.19224400
H	3.62750400	0.75915800	0.75568100
H	5.37626300	-2.41057700	-1.40795400
N	4.16420200	-1.05628700	-0.25104400
C	2.99553300	-1.90783200	-0.01776400
H	3.00490500	-2.69877800	-0.76839300
C	1.63281000	-1.17646800	-0.07488000
O	0.68286300	-2.03662900	-0.12295900
O	1.58267400	0.05272200	-0.03072800
C	-5.29983200	-0.27920000	2.13792700
H	-5.33195100	-1.29104100	2.54613400
H	-4.53705600	0.29787000	2.66213800
H	-6.28144300	0.19313400	2.21813100
C	-4.79248000	1.28904400	-0.11638900
C	-5.95841300	1.83212500	-0.67186800
C	-3.63445200	2.06263200	0.01384600
C	-5.97333500	3.16640400	-1.07977200
H	-6.84326200	1.21262400	-0.78800200
C	-3.66388100	3.39643200	-0.40020300
H	-2.71501700	1.64732200	0.41490100
C	-4.82507000	3.95016300	-0.94167600
H	-6.87752900	3.58873400	-1.51023500
H	-2.76152100	3.99195800	-0.29859600
H	-4.83540500	4.98808500	-1.26444500
H	5.66913100	2.30455700	1.04239600
H	3.07761000	-2.38220100	0.96589800
O	-0.55205400	1.73811500	1.10730100
H	0.15377000	1.41014100	0.52728000
H	-0.80549200	0.91419300	1.56164600

TS_a3

Mo	-1.34311500	-1.82043900	-0.24706000
O	-1.89293800	-3.18363700	-1.11093600
O	-1.18199900	-0.39048300	-1.41300500
O	-0.63575700	-2.33638200	1.50672500
O	-2.68967300	-0.34142600	-0.36701900
O	-2.04536600	-2.05142800	1.59017600
S	-4.17429500	-0.02778600	1.10718700
O	7.70274200	3.55518700	0.16776600
C	7.47876200	2.33572100	0.16811500
O	8.22206300	1.34027400	0.00170800
C	5.95380600	2.03466300	0.52622400
H	5.31155000	2.76126000	0.02791300
N	5.45789700	0.68545800	0.16866600
C	6.20022000	-0.48097400	0.21866800
C	4.22262100	0.39401800	-0.25188200
C	5.37602400	-1.48867800	-0.18764000
H	7.25123200	-0.40464800	0.45733600
H	3.39796200	1.07917100	-0.34476000
H	5.55630200	-2.54445600	-0.30821000
N	4.14259200	-0.92784700	-0.48029200
C	2.98591600	-1.65609900	-1.00420900
H	2.92030300	-1.51242400	-2.08783600
C	1.62850600	-1.23728400	-0.39115000
O	0.72671200	-2.09650200	-0.70180700
O	1.52759900	-0.19105500	0.24982600
C	-3.01549500	0.77247600	2.26357900
C	-4.88909000	1.36537100	0.25064200
C	-6.26768000	1.34342500	0.00327600
C	-4.10502200	2.43284300	-0.21023400
C	-6.86585500	2.39063500	-0.69832900
H	-6.86555900	0.51101500	0.36290400
C	-4.71784800	3.47965700	-0.89887200

H	-3.03224800	2.43950200	-0.04710600
C	-6.09316000	3.46355900	-1.14585400
H	-7.93558300	2.36908100	-0.88826100
H	-4.11065600	4.30720700	-1.25493100
H	-6.55922300	4.28201100	-1.68778100
H	-2.22158900	1.27077500	1.69809500
H	-3.55991500	1.46448500	2.91001100
H	-2.58511600	-0.04943200	2.83556100
H	3.14862900	-2.71842100	-0.82005100
H	5.83889100	2.15567000	1.60782000
O	-0.62175100	1.89802000	0.14673700
H	0.13705900	1.40526100	0.49583300
H	-0.93924400	1.26712800	-0.52901100

TS_a4

Mo	1.43335600	-1.59408100	-0.29592300
O	1.88261400	-3.21334800	-0.59730900
O	1.17396400	-1.28329300	1.50896200
O	0.90436100	-0.68269400	-1.94476000
O	2.86861700	-0.90226100	0.93138200
O	2.28825700	-0.44696700	-1.62954400
S	4.82208000	-0.43286100	0.23758100
C	5.37523100	-0.50338800	1.97356100
H	6.34475700	-0.01337500	2.08723700
H	4.61594100	-0.03223500	2.59895800
C	4.73495900	1.34670700	-0.01914900
C	3.57542400	2.06025100	0.30157400
C	5.84433400	1.99463700	-0.57759600
C	3.54749100	3.43833700	0.07541400
H	2.69635400	1.56640800	0.70454600
C	5.80205500	3.37199400	-0.79731200
H	6.72939000	1.42301100	-0.84229900
C	4.65284300	4.09497300	-0.46829400
H	2.64397000	3.98743800	0.32317600
H	6.66209400	3.87574500	-1.23061800
H	4.61795700	5.16694700	-0.64494200
O	-8.04270300	3.12611500	-0.43023200
C	-7.71211800	1.98041100	-0.09009900
O	-8.37217300	0.93174000	0.09875200
C	-6.15058000	1.90425800	0.22497100
H	-5.99160300	2.34023800	1.21614800
N	-5.55790900	0.54728700	0.22904800
C	-6.18653500	-0.60038100	0.67788000
C	-4.33196900	0.22945200	-0.20006300
C	-5.30306500	-1.62491600	0.50574600
H	-7.22190400	-0.53054300	0.97782400
H	-3.57809700	0.90865900	-0.55868500
H	-5.39473400	-2.67932800	0.70958200
N	-4.14727700	-1.09273500	-0.04451200
C	-2.97438400	-1.87109900	-0.45043100
H	-2.99979600	-2.81816800	0.08954700
C	-1.61229900	-1.18355100	-0.19298100
O	-0.66478600	-2.03850200	-0.31852900
O	-1.55836300	0.02287100	0.04769500
H	5.45234000	-1.56074400	2.23342900
H	-3.03664900	-2.09641700	-1.52032400
H	-5.60063000	2.49834300	-0.50528100
O	0.56742500	1.48497100	1.48846900
H	-0.15307500	1.24032300	0.88530100
H	0.84974900	0.60388200	1.79369100

TS_c1

Mo	-2.03834200	-1.60955700	-0.74091300
O	6.36423400	2.43596400	-2.54858600

C	6.43972700	1.44006300	-1.81035300
O	7.17763700	0.42316800	-1.85434700
C	5.50582200	1.53104700	-0.51971600
H	4.70229200	2.24729800	-0.71086700
C	6.35534500	1.98720200	0.69529600
H	7.21065800	1.29973300	0.73815200
C	6.88898200	3.40749900	0.44508900
H	7.37218800	3.48507000	-0.53014000
H	6.06834600	4.13556300	0.46388900
H	7.60260300	3.68857100	1.22790300
C	5.60672700	1.92831600	2.03519100
H	5.29280400	0.91264200	2.29774200
H	6.25026500	2.28880400	2.84563400
H	4.71285000	2.56511400	2.02024400
N	4.83092100	0.23690400	-0.23375200
C	5.40795800	-1.01805700	-0.32682200
C	3.56413900	0.09807600	0.17484400
C	4.45028900	-1.91919600	0.03626000
H	6.41183400	-1.08322600	-0.72335900
H	2.83539200	0.87933600	0.30679800
H	4.47613900	-2.99540400	0.08611400
N	3.30127100	-1.20955300	0.34684700
C	2.05033500	-1.79872900	0.86828800
H	2.09992800	-2.86446100	0.63463200
C	1.92808200	-1.60077100	2.40567000
H	1.89178500	-0.51588900	2.57190800
C	3.13870700	-2.17573500	3.15508900
H	3.20879500	-3.26145900	3.01258200
H	4.08493900	-1.73103500	2.83268700
H	3.03523200	-1.99228300	4.22945900
C	0.61944600	-2.21129800	2.93357400
H	0.56459400	-2.07562000	4.01895000
H	-0.26781800	-1.75417700	2.48826100
H	0.57881800	-3.28750600	2.72856300
C	0.81514500	-1.19135900	0.16154000
O	-0.03453900	-2.09002200	-0.18840600
O	0.72674000	0.03626400	0.06270600
O	-2.61595000	-3.21294100	-0.76770800
O	-1.09572800	-1.18141700	-2.40254300
O	-2.49678600	-0.86811200	-2.51804600
O	-2.10552900	-0.97764500	1.00744500
O	-3.45819000	-0.36914800	-0.06519500
O	-1.53153400	1.78913700	0.99011700
H	-1.88573700	0.89270800	1.15402600
H	-0.69790400	1.56517000	0.54851600
S	-4.74228900	0.68954700	-1.38507000
C	-5.65734500	1.46947900	-0.06606800
C	-7.05268900	1.34470500	-0.07854000
C	-5.01802700	2.14757000	0.98213600
C	-7.81139900	1.90310900	0.95052400
H	-7.53795400	0.81638800	-0.89406600
C	-5.78972700	2.71268300	1.99753600
H	-3.93556000	2.22052200	1.01372000
C	-7.18195800	2.59353200	1.98767000
H	-8.89344000	1.80379300	0.93596300
H	-5.29448100	3.23946500	2.80851400
H	-7.77263000	3.03396000	2.78639800
C	-3.47731700	1.93994800	-1.78112200
H	-2.91728800	1.51182100	-2.61246300
H	-3.96163900	2.87573200	-2.06872900
H	-2.80964800	2.06605000	-0.92322400

TS_c2

Mo	-2.19003300	-1.13764000	-0.72240000
O	-2.48787700	-2.73375900	-1.25273600
O	-1.83473500	-0.06863700	-2.38082500

O	-1.95690900	-1.19434800	1.10057500
O	-3.21000200	-0.00495700	-1.96334100
O	-3.68761800	-0.80814900	0.57099400
S	-5.63454300	-0.39249600	-0.08142200
O	6.59403600	3.31436600	-1.42892100
C	6.62660700	2.08150000	-1.28060800
O	7.24889400	1.18277500	-1.90148100
C	5.81664300	1.57150300	-0.00465900
H	5.08535700	2.33301400	0.27916200
C	6.80591400	1.31415400	1.16167000
H	7.57447900	0.64090700	0.75953000
C	7.48249000	2.63521900	1.56388700
H	7.89874400	3.15065100	0.69687200
H	6.75636000	3.31239000	2.03080500
H	8.28004600	2.44764600	2.29175400
C	6.15983700	0.65280700	2.38807900
H	5.74849700	-0.33714100	2.16501800
H	6.90051600	0.52427200	3.18552000
H	5.34913300	1.27293500	2.79159700
N	5.02729000	0.34680500	-0.30408900
C	5.44996900	-0.72581500	-1.07051100
C	3.79142000	0.09364700	0.14393200
C	4.43099000	-1.63307100	-1.07282900
H	6.40400600	-0.64364500	-1.57258400
H	3.16838400	0.74510900	0.73232100
H	4.34036500	-2.59183100	-1.55652600
N	3.39909800	-1.10900600	-0.31143400
C	2.13744000	-1.80957000	0.01225200
H	2.05549900	-2.62728000	-0.70699500
C	2.16247700	-2.37989700	1.45847200
H	2.23693700	-1.51241100	2.12794000
C	3.37725900	-3.28957400	1.69338700
H	3.34115500	-4.17003700	1.03953400
H	4.32874600	-2.77829100	1.51878300
H	3.37917400	-3.64982500	2.72729200
C	0.85532800	-3.12437600	1.77595800
H	0.90943800	-3.54000100	2.78807500
H	-0.02595100	-2.47975600	1.72011700
H	0.70183600	-3.95792900	1.08064400
C	0.91770400	-0.87306100	-0.16981500
O	-0.04104000	-1.42683300	-0.80323300
O	0.97376500	0.25911100	0.33689000
C	-6.26172800	-1.10321400	1.47539800
H	-6.25201300	-2.18726900	1.34767700
H	-5.57688900	-0.82592600	2.27771500
H	-7.27774000	-0.75742200	1.67893400
C	-5.74643800	1.36586100	0.28779900
C	-7.00216700	1.98737000	0.28892100
C	-4.58157300	2.10331200	0.52120700
C	-7.09645300	3.35391000	0.55174400
H	-7.89719000	1.40805000	0.07885200
C	-4.69110800	3.47446300	0.76990400
H	-3.61014000	1.61981000	0.50170400
C	-5.93880100	4.09903800	0.79196100
H	-8.07029700	3.83644400	0.55744000
H	-3.78627900	4.04946300	0.94407700
H	-6.01167300	5.16569500	0.98785000
O	-1.47224100	1.43015500	-0.17979700
H	-0.58134300	1.15914100	0.12711900
H	-1.40487700	1.42642900	-1.14991000

TS_c3

Mo	-2.07175100	-1.06690100	-0.38239600
O	-2.47749600	-2.20016400	-1.59144200
O	-2.03109500	0.60633700	-1.18190300
O	-1.42411700	-2.10976300	1.16735100

O	-3.63446400	0.19524400	-0.34857800
O	-2.82862800	-1.84409300	1.26706300
S	-5.32723500	-0.25884800	0.81967900
O	6.28139500	4.05810000	-0.03029500
C	6.37117300	2.93553100	-0.55449600
O	6.86875900	2.56218200	-1.64689400
C	5.85794700	1.75128700	0.38294200
H	5.18329400	2.16201400	1.13874500
C	7.07437300	1.06961000	1.06121600
H	7.75524500	0.79146300	0.24569700
C	7.79428500	2.08181900	1.96754100
H	8.01511200	3.00807500	1.43479500
H	7.16654800	2.34127800	2.82923200
H	8.72679700	1.65231700	2.35109800
C	6.71393600	-0.19386700	1.85688300
H	6.27294900	-0.97518200	1.22915400
H	7.61067400	-0.61825600	2.32233600
H	6.00404200	0.03298000	2.66280800
N	5.05460400	0.75552900	-0.37518300
C	5.34174400	0.27378100	-1.64087400
C	3.94106800	0.15981000	0.06867700
C	4.36672200	-0.62953100	-1.94814800
H	6.17191200	0.71572400	-2.17403400
H	3.43287700	0.34680200	0.99896300
H	4.20547800	-1.22009600	-2.83506400
N	3.49576900	-0.69143100	-0.87229500
C	2.34665800	-1.61563000	-0.76059300
H	2.14115700	-1.96138100	-1.77602700
C	2.68249600	-2.83145700	0.14858900
H	2.83900100	-2.42724300	1.15776200
C	3.96559800	-3.54734200	-0.29962600
H	3.85125600	-3.96207200	-1.30904300
H	4.83926300	-2.88896900	-0.30238600
H	4.18148700	-4.38226000	0.37482900
C	1.50581100	-3.82023500	0.19841700
H	1.77062900	-4.67095100	0.83549500
H	0.58913600	-3.37563900	0.59112500
H	1.28259700	-4.20962700	-0.80184600
C	1.08988400	-0.88053700	-0.22960000
O	0.03326000	-1.19824300	-0.87064100
O	1.22213100	-0.13055000	0.74958700
C	-4.59188600	0.51905600	2.29559500
C	-6.37373800	1.06226300	0.19094300
C	-7.74447700	1.03625900	0.47447800
C	-5.83366800	2.08355900	-0.60088000
C	-8.57561400	2.04307200	-0.02028700
H	-8.15524400	0.23091000	1.07643300
C	-6.67350100	3.08559200	-1.08936300
H	-4.77486400	2.06096600	-0.83555300
C	-8.04015300	3.06995600	-0.79995700
H	-9.63914400	2.02223300	0.20196500
H	-6.25700200	3.87769800	-1.70550400
H	-8.68755900	3.85234700	-1.18703600
H	-4.14030400	1.46801200	2.00713700
H	-5.34650000	0.64996700	3.07475600
H	-3.80007000	-0.16555200	2.60387800
O	-1.30168800	0.84857800	1.40270000
H	-0.35995800	0.58784000	1.34539000
H	-1.45852000	1.34032300	0.57867800

TS_c4

Mo	-2.17154400	-1.38335300	-0.47234400
O	-2.57987900	-2.93970400	-1.04397600
O	-1.79290600	-0.22094000	-1.85715900
O	-1.79965400	-1.39037100	1.45068400
O	-3.54033200	-0.17893300	-1.31474100

O	-3.17306200	-1.05902500	1.18086400
S	-5.56393100	-0.14963300	-0.65810400
C	-6.03214300	0.43111300	-2.32198100
H	-7.02766800	0.88035400	-2.30784600
H	-5.27963400	1.14254900	-2.66421700
C	-5.63376400	1.39243400	0.26968500
C	-4.46271100	2.09900500	0.56018600
C	-6.87835900	1.84974400	0.72385400
C	-4.55431900	3.28311200	1.29700900
H	-3.49033500	1.74578600	0.23212100
C	-6.95480900	3.03655400	1.45274700
H	-7.77789300	1.27795100	0.51262800
C	-5.79072500	3.75501600	1.73909300
H	-3.64148600	3.82807800	1.51812400
H	-7.92000100	3.39337700	1.80262300
H	-5.85023300	4.67701900	2.31176000
O	6.21155700	3.30245700	-1.91013900
C	6.33336700	2.10190000	-1.61694100
O	7.02162500	1.18415700	-2.13245800
C	5.56138700	1.68974800	-0.28262100
H	4.77281600	2.42190700	-0.08933000
C	6.56670700	1.65401000	0.89813500
H	7.38425300	0.99561400	0.57531800
C	7.14175700	3.06149300	1.12829000
H	7.51975300	3.49437500	0.20085700
H	6.36619600	3.73555700	1.51280300
H	7.94930700	3.02501300	1.86833300
C	5.97439600	1.10323900	2.20389200
H	5.62811400	0.06914200	2.10641100
H	6.72818900	1.11993900	2.99919000
H	5.12746500	1.71286000	2.54406100
N	4.86847800	0.38020800	-0.42481400
C	5.36189400	-0.73270500	-1.08409900
C	3.66565600	0.08183800	0.08143000
C	4.41885900	-1.71061700	-0.96433100
H	6.29875500	-0.62877000	-1.61463700
H	3.00353300	0.74189800	0.61524800
H	4.39679800	-2.71944600	-1.34256600
N	3.36229900	-1.19018000	-0.23390700
C	2.17580800	-1.95775400	0.20173300
H	2.12716800	-2.82960200	-0.45444200
C	2.31093400	-2.42318200	1.68019400
H	2.31525900	-1.51020000	2.29051300
C	3.62022300	-3.18726600	1.92702100
H	3.66113900	-4.10212100	1.32248800
H	4.50819400	-2.59129200	1.69788000
H	3.68380000	-3.48649400	2.97825200
C	1.10379600	-3.28007000	2.09637600
H	1.23002900	-3.60980900	3.13310800
H	0.15602900	-2.74312200	2.02505700
H	1.02748500	-4.17545500	1.46825400
C	0.87811200	-1.13054800	0.02000800
O	-0.07271400	-1.82949100	-0.48303100
O	0.86410700	0.04107300	0.41040000
H	-6.02343500	-0.44632300	-2.97126900
O	-1.27374000	1.97933700	-0.13100300
H	-0.55470300	1.44449200	0.24588200
H	-1.46301600	1.49059500	-0.95107700
